# Development of a functioning metric for the ageing population using data from the survey of health, ageing and retirement in Europe (SHARE)

**DOI:** 10.1371/journal.pone.0320068

**Published:** 2025-04-24

**Authors:** Carolina Fellinghauer, Jsabel Hodel, Beatriz Moreira, Jiin Kim, Carla Sabariego

**Affiliations:** 1 Swiss Paraplegic Research, Nottwil, Switzerland; 2 Faculty of Health Sciences and Medicine, University of Lucerne, Lucerne, Switzerland; 3 Center for Rehabilitation in Global Health Systems, University of Lucerne, Lucerne, Switzerland.; University of Copenhagen, DENMARK

## Abstract

**Background:**

Beyond mortality and morbidity, health statistics would benefit from reporting information on functioning, the third health indicator. The objective of this article is to use data from the Swiss Survey of Health, Ageing and Retirement in Europe (SHARE) to exemplarily create a psychometrically sound and valid metric of functioning for the ageing population living in Switzerland.

**Methods:**

Partial Credit Model (PCM) analysis, including analysis of targeting, item fit, local item dependencies (LID), unidimensionality, and differential item functioning (DIF), tested the psychometric properties of selected items. The DIF analysis investigated the invariance of item difficulties across sex and age groups, country, language, and the assessment Wave.

**Results:**

Data from 34,092 individuals aged 50 years and older was selected across assessment Waves of SHARE. The analysis showed that a functioning metric can be constructed with a total of 33 functioning items. Items showed LID and multidimensionality initially, which was solved with a testlet approach. Aggregation into testlets resulted in good fit, unidimensionality, no LID, and no DIF for sex, country, language, and the assessment Wave. Some DIF is found for age groups. The analysis also showed that the selected items target higher levels of problems in functioning than observed in the study population.

**Conclusions:**

A functioning metric can be constructed from selected functioning items of SHARE. The metric provides a sound interval-scaled score that can be used for longitudinal analyses of ageing in Switzerland and neighboring countries or as an indicator of the level of functioning in an ageing population.

## 1. Introduction

Health statistics typically report two health indicators: mortality and morbidity [1]. Mortality monitors the number of deaths in a population, e.g., due to a health event, and is communicated as a rate or an absolute number. Morbidity is an indicator of the prevalence or the incidence of health conditions in populations. Mortality and morbidity can be coded using the International Classification of Diseases (ICD) [2]. Due to current demographic and epidemiological trends, in particular the global ageing of populations and the increase in non-communicable diseases (NCDs) – the World Health Organization (WHO) has recommended the use of a third health indicator to complement mortality and morbidity, namely functioning [3]. Functioning can serve as an indicator of a population’s health state and inform public health systems steering and planning to achieve particular health goals [3].

The concept of human functioning was first introduced by WHO to describe what matters most to people about their health – in other words, how they experience health every day [4]. Formally, human functioning has been defined by WHO as the outcome of the interaction between health conditions and the physical, social and attitudinal environment of a person or population [5]. In a functioning perspective, health is not separated from what people can do, how they live, their behaviors and habits, socioeconomic resources and the environmental setting [6]. In this sense, functioning integrates biological health as a determinant and part of the actual everyday experience of health and is sensitive to the context of a person’s life. The importance of adopting a human functioning perspective when talking about health becomes clear when considering the extent to which assistive technologies and accessible environments can support persons with a health condition in performing everyday activities and participating in the society [7].

The International Classification of Functioning, Disability, and Health (ICF) encompasses the categories and definitions to describe functioning and health comprehensively. Over the years, the ICF has been used as a reference framework to link and summarize qualitative information and to guide the collection, analysis and reporting of data on functioning. One way to obtain data on functioning is by directly rating the extent of problems in relevant ICF categories, for instance self-care or mobility [8,9]. However, operationalizing relevant ICF categories as questions improves the quality of the data collected. Corresponding functioning assessment tools can be developed by creating items *ab initio* or with the help of existing questions from extensively tested and validated assessment instruments [10,11]. For the latter, functioning data can be identified in surveys or health assessments by linking them to relevant ICF categories so that a functioning metric can be built [12,13]. In this context, we systematically use the term “metric” as a synonym of the generated functioning scale that, in our case, has cardinal properties and ranges from zero (worst functioning) to 100 (best functioning). We use the term “score” when writing about the psychometrically-derived 0–100 functioning score of a person that can be located on the functioning scale. The term (functioning) indicator, in this context, would be a summary statistic (e.g., mean, median, quantiles) for a population calculated with the functioning scores.

The use of solid statistical methods in the development of a valid and reliable functioning metric is essential. It can solidify the role of functioning as the third health indicator in the health care system. Common statistical approaches to obtain a sound functioning metric use modern test theory methods. For example, data on functioning that was rated on an ordinal response scale (e.g., How much of a problem is it for you to…? 1 = no problem, 2 = mild problem, 3 = moderate, etc.) can be calibrated using Item Response Theory (IRT) to derive interval-scaled estimates of functioning [14], for measurement at the level of the individual or populations. Psychometric analyses of functioning data and the creation of a functioning metric as the basis for a corresponding functioning indicator have already been undertaken in various studies. The Model Disability Survey (MDS) is a general population survey initiated by WHO that is suitable for describing the functioning and disability levels of populations in various countries and regions [15]. The detailed information that the MDS collects enables the quantification of functioning of people with different health conditions, helps to define groups experiencing mild, moderate or severe disability, and to identify their specific needs, barriers, and inequalities. A shorter version of the MDS, the Brief MDS, can be integrated into existing and regularly conducted household surveys to obtain a functioning metric [11]. The WHO’s Functioning and Disability Disaggregation Tool (FDD11) is another functioning and disability assessment tool consisting of 11 essential items necessary to obtain a reliable functioning metric [16]. Despite the availability of functioning-based tools, constructing a functioning metric is not conditional on having specific tools. In an exemplary study, Cieza and colleagues used psychometric methods to show how functioning information collected with self-reported questions and physical measurements in two ageing cohort studies, using common items, enabled comparisons of functioning and health across populations of persons with different health conditions in the United Kingdom and in the United States [17]. In the field of spinal cord injury (SCI) rehabilitation, two studies in Switzerland showed that data collected during and after rehabilitation using a range of different tools, and analysed with Rasch methods, provided information on the functioning of this particular population. It was also possible to derive typologies of changes in functioning over time [18, 19]. In another study, a comparative cross-country exploration, used a similar approach to unveil differences in the functioning of persons with SCI living in Europe [20]. IRT methods and self-reported questions from different tools implemented in cohort studies have also been prominently used in ageing research to model and examine trends in functioning and morbidity over time [21].

An extensive and systematic analysis of the availability of functioning data in Switzerland recently identified and compared data collected in the Swiss part of the Survey of Health, Aging, and Retirement in Europe (SHARE), the Swiss Health Survey, the Lausanne Cohort 65+, and the Swiss Household Panel [13]. Although functioning data is collected in diverse surveys in Switzerland, the construction of a functioning metric for the ageing population has not been undertaken yet. Consequently, statistical modeling of functioning over time and its use as a health indicator is limited. Therefore, the objective of this study is to use items of SHARE that were previously identified and linked to the ICF [13], i.e., items that ask about or describe the extent of a functioning problem, to construct a sound metric of functioning with interval scale properties usable for individual- and population-level statistics.

## 2. Methods

### 2.1. Sample and procedure

SHARE is a multidisciplinary and cross-national panel survey that has been conducted biannually since 2004 [22]. The survey collects health, socioeconomic status, and social and family network data. The SHARE target population consists of persons aged 50 years and older at the time of sampling who have their regular domicile in the respective SHARE country. Persons are excluded if incarcerated, hospitalized, out of the country during the entire survey period, do not speak the respective country’s language(s), or have moved to an unknown address. Age-eligible respondents who participated are followed and re-interviewed if they move within the country. Persons living in a nursing home or another institution for the elderly are part of the target population investigated by SHARE but may not be equally represented in all countries depending on the sampling frame coverage. With the public release of Wave 8 in spring 2022, the data available to the scientific community consists of more than 530’000 interviews with 140,000 survey participants, and covers all 26 continental European Union Member states as well as Switzerland and Israel.

### 2.2. Study design

This psychometric study uses data from SHARE Waves 1, 2, 4, 5, 6, 7, and 8 [23–28]. See Börsch-Supan et al. [29] for methodological details and Bergmann et al. [30] for an overview of response and retention rates.

**Fig 1:**
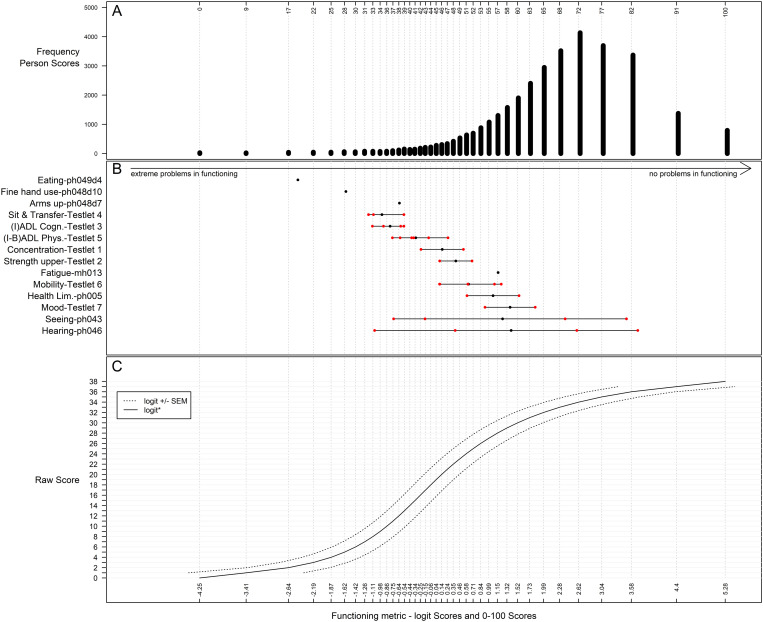
Person-Item Map after adjustments (testlet solution), with a histogram for the frequencies of the functioning levels for the analysis population in panel A, the mean difficulty (black dots) and response option thresholds (red dots) of the items/testlets and assessment ranges in logits and transformed to a 0-100 interval-scaled score in panel B, and the score to estimate functioning with standard error of measurement (SEM) in panel C. Lower functioning scores indicate lower levels of functioning (i.e., higher disability) and higher scores indicate higher levels of functioning (i.e., lower disability).

The analysis used data from Switzerland and four neighboring countries: Germany, France, Italy, and Austria. All five countries have participated in the SHARE survey since 2004. The SHARE variables selected for this study consisted of socio-demographic and sample characteristics as well as items that ask for the presence or the intensity of a functioning problem [13]. The latter was used to construct the functioning metric in line with the ICF definition of functioning, an umbrella concept that encompasses body functions, such as sleep or cardiovascular functions; activities, such as walking or self-care; and participation domains, such as working or engaging in social activities. Data used to construct a functioning metric must include information about two components: body functions, and activities and participation. In the absence of a functioning-specific questionnaire, a selection of items or physical measurements addressing problems in body functions, and activities and participation would be used to build a metric, as illustrated by the aforementioned work published by Cieza and colleagues [17]. In the present study, we used a systematic mapping of SHARE by Moreira and colleagues [13] to inform the selection of the items suitable to build a functioning metric. In total, 33 items assessing components of functioning were retained, with nine items assessing body functions and 28 items asking about problems in activities and participation (see Table 1 for the ICF linking of the selected functioning items in SHARE). The analysis did not include individuals with more than 30% missing values. Details on percentages of missing values and availability per SHARE assessment Waves, per country, and per item are shown in the supplementary material as tables (S1 Table 1–6 in S2 File) and figures (S1 Fig 1–6).

**Table 1:** SHARE items selected to build the metric of functioning, the linked ICF categories and item response options as assessed in SHARE.

Item identifier	Label	ICF-linking	Rating in SHARE
ph005	Limited in activities because of health	d230	1=Severely limited; 2=Limited, but not severely; 3=Not limited
ph043	Eyesight distance	b21000	1=Excellent; 2=Very good; 3=Good; 4=Fair; 5=Poor
ph044	Eyesight reading	b21002	1=Excellent; 2=Very good; 3=Good; 4=Fair; 5=Poor
ph046	Hearing	b230	1=Excellent; 2=Very good; 3=Good; 4=Fair; 5=Poor
ph048d1	Difficulties: walking 100 metres	d4500	0 = Not selected; 1=Selected
ph048d2	Difficulties: sitting two hours	d4153	0 = Not selected; 1=Selected
ph048d3	Difficulties: getting up from chair	d4104	0 = Not selected 1=Selected
ph048d4	Difficulties: climbing several flights of stairs	d451	0 = Not selected; 1=Selected
ph048d5	Difficulties: climbing one flight of stairs	d451	0 = Not selected; 1=Selected
ph048d6	Difficulties: stooping, kneeling, crouching	d4105, d4102, d4101	0 = Not selected; 1=Selected
ph048d7	Difficulties: reaching or extending arms above shoulder	d4458	0 = Not selected; 1=Selected
ph048d8	Difficulties: pulling or pushing large objects	d445	0 = Not selected; 1=Selected
ph048d9	Difficulties: lifting or carrying weights over 5 kilos	d430	0 = Not selected; 1=Selected
ph048d10	Difficulties: picking up a small coin from a table	d440	0 = Not selected; 1=Selected
ph049d1	Difficulties: dressing, including shoes and socks	d540	0 = Not selected; 1=Selected
ph049d2	Difficulties: walking across a room	d4600	0 = Not selected; 1=Selected
ph049d3	Difficulties: bathing or showering	d510	0 = Not selected; 1=Selected
ph049d4	Difficulties: eating, cutting up food	d550	0 = Not selected; 1=Selected
ph049d5	Difficulties: getting in or out of bed	d410	0 = Not selected; 1=Selected
ph049d6	Difficulties: using the toilet, incl getting up or down	d530	0 = Not selected; 1=Selected
ph049d7	Difficulties: using a map in a strange place	b1141	0 = Not selected; 1=Selected
ph049d8	Difficulties: preparing a hot meal	d630	0 = Not selected; 1=Selected
ph049d9	Difficulties: shopping for groceries	d6200	0 = Not selected; 1=Selected
ph049d10	Difficulties: telephone calls	d3600	0 = Not selected; 1=Selected
ph049d11	Difficulties: taking medications	d5702	0 = Not selected; 1=Selected
ph049d12	Difficulties: doing work around the house or garden	d640, d6505	0 = Not selected; 1=Selected
ph049d13	Difficulties: managing money	d860	0 = Not selected; 1=Selected
mh002	Sad or depressed last month	b152	1=Yes; 5=No
mh007	Trouble sleeping	b134	1=Trouble with sleep or recent change in pattern; 5=No trouble sleeping
mh010	Irritability	b1263	1=Yes; 5=No
mh013	Fatigue	b1300	1=Yes; 5=No
mh014	Concentration on entertainment	d160, b140	1=Difficulty in concentrating; 5=No such difficulty mentioned
mh015	Concentration on reading	d160	1=Difficulty in concentrating; 5=No such difficulty mentioned

Note: SHARE, Survey of Health, Ageing and Retirement in Europe.

Five additional functioning items that would have been eligible were not included in the analysis due to the large amount of missing values (>60%) or for not having been assessed in the earlier SHARE Waves. These items were ph084 - Trouble with pain (66.7% missing), ph085 - Level of pain (85.1% missing), ph049d14 - Leaving the house independently (67.3% missing), ph049d15 - Doing personal laundry (67.3% missing), and cf103 - Memory (69.1% missing). The two pain items (ph084 and ph085) would have to be combined to make a single pain item, as the pain level is only asked if the response to ph084 is yes. A preliminary Rasch analysis, solely with Wave 8 data and keeping the aforementioned excluded items, showed misfit in the items assessing pain (ph085) and memory (cf103). The items ph049d14 and ph049d15 had high correlations with the items ph049d3, ph049d8, ph049d9, ph049d12, and ph049d13, supporting some redundancy in the content assessed by these two items with other items from the functioning metric. The removal of these items had a slightly positive effect on the infit and outfit values of the metric in general, and the remaining 33 items were expected to provide sufficient to good coverage of the functioning construct.

### 2.3. Psychometric analysis

Noteworthy about this study is that it does not use an established functioning questionnaire but rather selected items from SHARE that assess problems in body functions, and activities and participation. Previous studies have confirmed the suitability of IRT models for the psychometric analyses of functioning information that is assembled from a survey or cohort study to construct a scale [17,20]. IRT models represent a class of psychometric model of different complexity and purposes which are based on a probabilistic approach to measurement [31]. One type of IRT models used to test the measurement properties of assessment tools or sets of items are the so-called Rasch models, which assume that responses to tests or assessments can be explained by the difficulty of the items (i.e., how easy or demanding an activity or task is according to the number of respondents who report it as a problem) and the level of functioning of the respondents (i.e., how many functioning problems people report in a given set of items). The items that were included in the analysis were mostly dichotomously-rated items (0 = Not Selected, 1 = Selected); three items had 5 options (1 = Excellent, 2 = Very good, 3 = Good, 4 = Fair, 5 = Poor), and one item had 3 response options (1 = Severely limited, 2 = Limited, but not severely, 3 = Not limited). Table 1 shows the SHARE items used to construct the functioning metric. Note that the selected items could also be coded as “Refusal” (-2) or “Don’t know” (-1), which were recoded as missing prior to the analysis. If applicable response options were recoded prior to analysis, where appropriate, so that higher ratings indicated higher functioning levels. An R-script for the recoding of variables, as used in this study, is found in the (S1 File). This study used the Partial Credit Model (PCM), an extension of the classical Rasch model for dichotomous data that can handle ordinal-scaled response options and items with varying numbers of response options [32].

Other IRT models, also found helpful in developing metrics of functioning with polytomous response options, are from the group of so-called “2-parameter logistic (2-PL) models”; these models take into account not only the difficulty of items but also the different levels of discrimination or “sharpness” of the items [31]. The main difference between the Rasch models (classical and PCM) and the 2-PL model is that in the former, the total score provides sufficient information to estimate the item difficulties, whereas the latter relies on the response patterns to obtain item parameter estimates. While our analysis of the SHARE data used both the PCM and the 2-PL model, we only kept the results of the PCM analysis. First, the analysis with 2-PL did not perform notably better in terms of item fit and second, raw scores can only be converted into interval-scaled scores without further ado if the fit to the PCM is confirmed.

When doing an analysis based on a Rasch model, several measurement assumptions are tested to determine if the data at hand fit the model and thus support the validity of a metric and respective derived scores for measurement [33]. First, the assessment tool has to show good targeting, meaning that the item difficulties have to match the abilities of the population. Second, with good targeting comes good person separation, which expresses how reliably the assessment tool can determine levels of the trait being measured (e.g., functioning). The Cronbach Alpha and the Person Separation Index (PSI), interpreted similarly to the Cronbach Alpha, provide information about the reliability. PSI values of minimum 0.7 are generally recommended to use the metric for measurement at group level. Root mean square error (RMSE) and percent bias are also discussed. The RMSE is always positive and values close to zero indicate that the response probabilities derived from the Rasch model perfectly describe the observed item responses. The percent bias indicates the average tendency of the observed scores to be larger or smaller than the expected scores. Forero and Maydeu-Olivares [34] recommend treating absolute percent bias values below 10% as negligible, values of 10% to 20% as substantial, and >20% as unacceptable. RMSE and percent bias were computed using the response probabilities obtained from the Rasch model. For each participant the expected sum score was determined on the basis of the most likely response pattern. The expected sum scores were compared to the sum scores found in the raw data. Computation of the most likely response pattern left out extreme scores from the original dataset, as response probabilities were not available. The sum scores were transformed to a 0–100 interval scale.

Third, items of the assessment tool are expected to present a good fit, have ordered response thresholds, and be free of local item dependencies (LID). Good item fit is determined using the Infit Mean Square (MSQ) and Outfit MSQ statistics, with values above 1.2 indicating underfit [35]. Small Infit and Outfit MSQ statistics (<0.5) are not productive but also not detrimental for measurement. Identifying LID includes checking the correlations among the standardized residuals of the Rasch analysis. A corresponding cut-off value is calculated which controls for the length of the assessment and would retain the residual correlations with values of 0.2 above the mean residual correlations [36]. Fourth, the residuals are expected to be free of any pattern that indicates the clustering of items or the presence of different dimensions. Accordingly, the unidimensionality of the residuals is investigated using a principal component analysis (PCA). A first eigenvalue above 2.00 is indicative of multidimensionality [37]. Lastly, items are expected to be equally difficult across relevant subgroups of the population under study. The corresponding analysis of differential item functioning (DIF) tested the invariance of the item difficulties across sex (male vs. female), age groups (from 50 to 100 years in 5-year increments), country (Switzerland, France, Germany, Italy, and Austria), language (German, French, and Italian), and assessment Wave by means of ordinal regressions [38]. DIF is seen as harmful for a tool if the differences across subgroups in responding to an item suggest that the item is significantly biased towards one group and that this bias indicates unfair assessment.

In preparation for the Rasch analysis, the data of individuals with less than 30% of the items missing per assessment Wave was imputed using a random forest-based approach [39]. Furthermore, to avoid potential bias in the PCM analysis due to repeated assessments, only one assessment Wave was included per individual, with a random selection from the available individual assessments across SHARE Waves, [40]. The Rasch analysis was conducted iteratively until the data fitted the model. The logit-scaled person parameter estimates derived by the final Rasch analysis were rescaled to represent 0–100 scores, where lower scores represent lower levels of functioning (i.e., higher levels of disability, more problems in functioning) and higher scores represent higher levels of functioning (i.e., lower levels of disability, less problems in functioning). Scores were then derived for the individual SHARE assessments across Waves and for the five countries. The raw scores of the final metric and the corresponding interval-scaled scores from the Rasch analysis are presented in a transformation table. The precision of the logit-scaled person parameter estimates shown in the transformation table is given by the standard error of measurement (SEM) [41]. The precision is expected to diminish in the extremes of the score continuum. The SEM is given in logits and transformed on a 0–100 scale, based on the assessment range of the metric. Finally, minimal important change (MIC) between Waves was calculated using the Reliable Change Index for IRT (RCIIRT) [42]. The RCIIRT observes a standardized change in the person parameter estimates across time points and has the advantage of using their local precision. Unlike other statistical estimates of MIC, the RCIIRT does not assume that the measurement error is constant across the measurement continuum. For this study, individual absolute RCIIRT values above 1.96 indicate reliable significant differences (5%**-**level), i.e., not due to random variations [43].

Analyses were performed using R software version 4.3.2 [33] and specifically the packages missRanger [34] for data imputation, mirt [44] for psychometric analyses, and lordif [28] for DIF analysis.

## 3. Results

### 3.1. SHARE sample

The original sample size was N = 110,436 with data from 35,862 unique individuals above 50 years of age, who could have participated in SHARE up to 7 times. After removing observations with more than 30% missing values, a large sample of N = 109,792 remained. From this starting sample, one unique observation from each participating individual was randomly selected to constitute the sample for the psychometric analysis. A first round of analyses was undertaken with this sample. Analysis of the fit of the person parameters indicated some strong outliers (N = 1,616), with fit values detrimental for analysis [45], which were removed from the sample. We therefore report the results of the psychometric analysis based on the remaining sample of N = 34,092 individuals. A sensitivity analysis was conducted to detect common characteristics specific to individuals misfitting the Rasch model, such as, for example, a higher prevalence of participants from a sex or age group, more participant data from a certain Wave or country, as well as unexpected characteristics of the response patterns. The sensitivity analysis did not unveil any systematic cause for the person misfit.

Table 2 presents the characteristics of included participants with the following sample sizes per country: Austria N = 5,874, France N = 7,526, Germany N = 8,279, Italy N = 8,063, and Switzerland N = 4,350. The percentages of male and female participants differed across countries, with the smallest percentage of male participants in Austria, i.e., 42.5%, and the largest percentage of male participants in Germany, i.e., 47.1%. The mean age was about 67 years across all the included countries, with the highest mean age in Austria, with 67.84 years (SD = 10.04), and the lowest age in Germany, with 66.12 years (SD = 10.03). Less than 10% of the participants in each country were older than 85 years of age. The proportion of married persons was lowest in Austria (69.1%) and above 75% in the other countries. In Austria, the proportion of widowed persons was 20%. In Switzerland, 23.6% of the respondents were French-speaking, 72.6% German-speaking, and 3.8% Italian-speaking. The other countries conducted assessments only in their main official language. The country with the highest mean years of education was Germany (12.28 years, SD = 2.54) and while Switzerland had the lowest (8.79 years, SD = 3.72). The percentages of individuals sampled per Wave differed across countries. The most frequently sampled individual data came from Wave 4 for Austria (31.6%), France (26.1%), and Switzerland (28.2%). In Germany, most of the sampled individual data came from Wave 1 (20.4%) and Wave 5 (27.8%), while most of the sampled individual data for Italy came from Wave 6 (21.3%).

**Table 2:** Descriptive statistics of the included population per country (N = 34,092).

	Austria	France	Germany	Italy	Switzerland
n	5874	7526	8279	8063	4350
Sex = Male (%)	2495 (42.5)	3365 (44.7)	3897 (47.1)	3716 (46.1)	2003 (46.0)
Age group (%)					
(50,55]	620 (10.6)	1226 (16.3)	1292 (15.6)	1085 (13.5)	556 (12.8)
(55,60]	849 (14.5)	1268 (16.9)	1390 (16.8)	1281 (15.9)	695 (16.0)
(60,65]	1010 (17.2)	1242 (16.5)	1379 (16.7)	1354 (16.8)	711 (16.3)
(65,70]	1046 (17.8)	979 (13.0)	1369 (16.5)	1315 (16.3)	714 (16.4)
(70,75]	893 (15.2)	882 (11.7)	1134 (13.7)	1143 (14.2)	592 (13.6)
(75,80]	681 (11.6)	790 (10.5)	876 (10.6)	963 (11.9)	457 (10.5)
(80,85]	464 (7.9)	624 (8.3)	523 (6.3)	556 (6.9)	358 (8.2)
(85,90]	214 (3.6)	333 (4.4)	217 (2.6)	241 (3.0)	197 (4.5)
>90	96 (1.6)	175 (2.3)	96 (1.2)	121 (1.5)	69 (1.6)
Age - mean (SD)	67.84 (10.0)	66.92 (11.13)	66.12 (10.03)	66.97 (10.15)	67.43 (10.48)
Marital Status (%)					
Divorced	340 (5.8)	332 (4.4)	360 (4.3)	132 (1.6)	214 (4.9)
Married	4056 (69.1)	5660 (75.2)	6513 (78.7)	6406 (79.4)	3424 (78.7)
Never married	221 (3.8)	292 (3.9)	268 (3.2)	311 (3.9)	113 (2.6)
Registered partnership	11 (0.2)	25 (0.3)	16 (0.2)	87 (1.1)	9 (0.2)
Separated	44 (0.7)	61 (0.8)	72 (0.9)	41 (0.5)	46 (1.1)
Widowed	1202 (20.5)	1156 (15.4)	1050 (12.7)	1086 (13.5)	544 (12.5)
Language (%)					
French	0 (0.0)	7526 (100.0)	0 (0.0)	0 (0.0)	1025 (23.6)
German	5874 (100.0)	0 (0.0)	8279 (100.0)	0 (0.0)	3159 (72.6)
Italian	0 (0.0)	0 (0.0)	0 (0.0)	8063 (100.0)	166 (3.8)
Years of education - mean (SD)	9.72 (3.00)	11.26 (2.74)	12.28 (2.54)	9.15 (3.68)	8.79 (3.72)
Wave (%)					
SHARE 1	630 (10.7)	1225 (16.3)	1687 (20.4)	1012 (12.6)	391 (9.0)
SHARE 2	354 (6.0)	958 (12.7)	1120 (13.5)	962 (11.9)	490 (11.3)
SHARE 4	1854 (31.6)	1961 (26.1)	410 (5.0)	1093 (13.6)	1228 (28.2)
SHARE 5	1171 (19.9)	1126 (15.0)	2305 (27.8)	1469 (18.2)	725 (16.7)
SHARE 6	808 (13.8)	1003 (13.3)	1138 (13.7)	1720 (21.3)	626 (14.4)
SHARE 7	752 (12.8)	701 (9.3)	944 (11.4)	1297 (16.1)	510 (11.7)
SHARE 8	305 (5.2)	552 (7.3)	675 (8.2)	510 (6.3)	380 (8.7)

Note: SD, standard deviation; SHARE, Survey of Health, Ageing and Retirement in Europe.

More than 20% of participants reported some difficulties with ph005 - Limited activities because of health (45.26%), mh002 - Sad and depressed in the last month (40.18%), mh013 - Fatigue (33.05%), mh007 - Having trouble sleeping (32.02%), ph048d6 - Stooping, kneeling, crouching (29.81%), mh010 - Irritability (27.87%), ph048d4 - Climbing several flights of stairs (26.94%), as well as ph048d9 - Lifting or carrying weights over 5 kilos (20.22%). More than 50% reported having some problems (i.e., not having excellent or very good performance) with ph046 - Hearing (59.34%), ph044 - Eyesight reading (58.12%), ph043 - Eyesight distance (53.53%), despite the use of assistive devices such as glasses and hearing aids. The full list of response frequencies and corresponding percentages for each functioning item and Wave for the total sample and by country are shown as part of the (S2 File).

### 3.2. Functioning metric

The results of an initial Rasch analysis showed that, at start, the set of selected items (Table 1) contained some misfitting items, as well as some content redundancies in form of LID and multidimensionality (see Table 4). Furthermore, DIF for age was observed in some items. After the initial analysis, two items were removed from the set of selected items, namely ph044 - Eyesight reading and mh010 - Irritability, as they did not fit the model and were locally dependent (LID) on other items of the scale (see the Supporting Information S1 Fig.). These two items were excluded, as attempts to solve the LID resulted in misfit in the testlets that included these items. Misfit indicates that items do not discriminate between different levels of functioning.

**Table 3:** Targeting, person separation index (PSI), and internal consistency of the functioning metric for the initial analysis and after adjustments for local item dependencies (LID) and multidimensionality (testlet solution).

	Initial analysis	Testlet solution
Item difficulties - mean (SD)	‒0.48 (1.82)	0.05 (1.34)
Person abilities - mean (SD)	2.18 (1.47)	2.09 (1.33)
Person Separation Index (PSI)	0.85	0.77
Cronbach Alpha	0.88	0.85

Note: SD, standard deviation; PSI, person separation index.

**Table 4:** Item fit, mean difficulty, difficulty range, local item dependencies (LID), and differential item functioning (DIF) for age groups for the functioning metric for the initial analysis.

Item Identifier	Outfit	Infit	Difficulty - mean (SD)	LID	Age grp. DIF
ph005	0.89	0.87	1.16 (0.79)		
ph043	1.17	1.14	1.24 (2.27)	ph044	
ph044	*1.48*	*1.39*	1.71 (1.93)	ph043	
ph046	*1.35*	*1.29*	1.42 (2.41)		*
ph048d1	0.47	0.76	‒0.59		
ph048d2	0.91	1.07	‒0.68		*
ph048d3	0.75	0.9	0.19		
ph048d4	0.76	0.83	0.89		
ph048d5	0.51	0.78	‒0.41		
ph048d6	0.8	0.84	1.08		
ph048d7	0.72	0.91	‒0.65		
ph048d8	0.53	0.78	‒0.27	ph048d9	
ph048d9	0.67	0.81	0.39	ph048d8	
ph048d10	0.72	0.97	‒1.72		
ph049d1	0.47	0.78	‒1.04		
ph049d2	0.18	0.72	‒2.59		
ph049d3	0.28	0.67	‒1.35	ph049d9	
ph049d4	0.26	0.75	‒2.7		
ph049d5	0.28	0.75	‒2.11	ph049d6	
ph049d6	0.27	0.72	‒2.47	ph049d5	
ph049d7	0.68	0.83	‒1.07		
ph049d8	0.26	0.68	‒1.95	ph049d9	
ph049d9	0.28	0.67	‒1.22	ph049d8; ph049d12; ph049d3	*
ph049d10	0.25	0.73	‒2.7		
ph049d11	0.19	0.68	‒2.61		
ph049d12	0.44	0.74	‒0.53	ph049d9	
ph049d13	0.4	0.75	‒1.88		
mh002	*1.3*	1.08	1.68	mh010	*
mh007	*1.42*	1.15	1.21		*
mh010	*1.93*	*1.36*	0.95	mh002	*
mh013	0.93	0.91	1.28		
mh014	1	1.01	‒0.33	mh015	
mh015	0.9	0.98	‒0.19	mh014	

Note: LID, local item dependency; DIF, differential item functioning; grp., group; SD, standard deviation.

The remaining items presented some LID with residual correlations above the study-specific cut-off. LID was solved using a stepwise creation of testlets until LID was no longer present. This resulted in seven testlets that aggregated items as follows:

- Testlet 1 (Concentration): mh014 - Concentration on entertainment, mh015 - Concentration on reading
- Testlet 2 (Upper body strength): ph048d8 - Pulling or pushing large objects, ph048d9 - Lifting or carrying weights over 5 kilos
- Testlet 3 (Instrumental activities of daily living, IADL, [46]): ph049d7 - Using a map to get around in a strange place, ph049d10 - Telephone calls, ph049d11 - Taking medications, ph049d13 - Managing money
- Testlet 4 (Sit and Transfer): ph048d2 - Sitting two hours, ph049d5 - Getting in or out of bed, ph049d6 - Using the toilet, incl getting up or down
- Testlet 5 (Basic and Instrumental ADLs): ph049d1 - Dressing, including shoes and socks, ph049d2 - Walking across a room, ph049d3 - Bathing or showering, ph049d8 - Preparing a hot meal, ph049d9 - Shopping for groceries, ph049d12 - Doing work around the house or garden
- Testlet 6 (Mobility): ph048d1 - Walking 100 metres, ph048d3 - Getting up from chair, ph048d4 - Climbing several flights of stairs, ph048d5 - Climbing one flight of stairs, ph048d6 - Stooping, kneeling, crouching
- Testlet 7 (Mood): mh002 - Sad or depressed last month, mh007 - Trouble sleeping

Tables 3–5 provide more information about the specific fit statistics of the initial analysis and the testlet solution presented after removing the two items mentioned above.

**Table 5:** Item identifier and descriptions, item fit, mean difficulty, difficulty range, minimum and maximum thresholds for items with more than two response options for the functioning metric after adjustments for local item dependencies (LID) and multidimensionality (testlet solution).

Item Identifier	Description	Outfit	Infit	Difficulty - mean (SD)	Min Threshold	Max Threshold	Age grp. DIF
ph049d4	Eating	0.23	0.75	‒2.47			
ph048d10	Fine Hand Use	0.55	0.88	‒1.6			
ph048d7	Arms up	0.6	0.84	‒0.63			
Testlet 4	Sit and Transfer	0.58	0.76	‒0.95 (0.35)	‒1.19	‒0.55	
Testlet 3	(I)ADL - cognitive	0.54	0.69	‒0.8 (0.27)	‒0.92	‒0.55	*
Testlet 5	(I-B)ADL - physical	0.32	0.52	‒0.33 (0.36)	‒0.75	0.25	*
Testlet 1	Concentration	1.07	1.09	0.15 (0.54)	‒0.24	0.53	
Testlet 2	Strength upper body	0.54	0.72	0.4 (0.41)	0.1	0.69	
mh013	Energy	0.83	0.87	1.16			
Testlet 6	Mobility	0.64	0.69	0.63 (0.53)	0.1	1.22	
ph005	Limitation due to health	0.74	0.77	1.07 (0.67)	0.6	1.54	
Testlet 7	Mood	*1.27*	1.15	1.38 (0.64)	0.93	1.84	*
ph043	Seeing	1.15	1.16	1.24 (2.02)	‒0.73	3.49	
ph046	Hearing	1.15	1.14	1.4 (2.15)	‒1.08	3.7	*

Note: Min, minimum, Max, maximum; DIF, differential item functioning; grp., group; SD, standard deviation.

The presence of LID in the initial analysis is reflected in the outcome of the dimensionality analysis, with multidimensionality and a first eigenvalue of 3.05 for the initial PCA analysis (see Table 4). The testlet solution supported unidimensionality with a first eigenvalue of 1.84 (see Table 5).

The results of the initial analysis, shown in Table 4, indicated item misfit in a few items, namely ph044 - Eyesight reading (Outfit MSQ = 1.48, Infit MSQ = 1.39), ph046 - Hearing (Outfit MSQ = 1.35, Infit MSQ = 1.29), mh007 - Trouble sleeping (Outfit MSQ = 1.42, Infit MSQ = 1.15), mh010 - Irritability (Outfit MSQ = 1.93, Infit MSQ = 1.36). The testlet solution could solve the LID and presented Infit MSQ values indicative of a good fit (see Table 5). The Outfit MSQ, which is more sensitive to outliers, was above the cut-off (i.e., 1.2) for Testlet 7 that aggregated the items mh002- Sad or depressed last month, mh007 - Trouble sleeping (Outfit MSQ = 1.27).

The initial Rasch analysis indicated DIF only for some items based on the comparison across age groups: ph046 - Hearing, ph048d2 - Sitting two hours, ph049d9 - Shopping for groceries, mh002 - Sad or depressed last month, mh007 - Trouble sleeping, and mh010 – Irritability (see Table 4). For the testlet solution, DIF for age groups was found for ph046 – Hearing, Testlet 3, the Testlet 5, and the Testlet 7. In all these settings, older age was associated with more problems in functioning. This is not surprising and does not necessarily indicate bias in the sense of an unfair assessment. No DIF was observed for sex, language, country, and the assessment Wave (see Table 5).

In general, at the level of the metric, the testlet solution showed sufficient to good reliability (PSI = 0.77, Cronbach alpha = 0.85), indicating that the developed functioning metric can be recommended for group-level measurement and analysis of changes over time. The RMSE was 6.56, indicating that on a 0–100 scale, the expected mean imprecision would be around ±6.5 units. The percent bias was negligible at 8.27%. The analysis of targeting, which should ideally show a match between the mean and dispersion of the item and the person parameter estimates, indicated that the SHARE items selected to assess functioning are targeted towards higher levels of disability than those observed in the study population (Table 3). The distribution of the person parameter estimates is strongly left-skewed, and item parameter estimates are missing where the density of person parameter estimates is highest.

This is also graphically shown in Fig 1, depicting the Person-Item Map for the testlet solution. The figure shows, at the top (panel A), a histogram of the distribution (frequencies) of the functioning levels for the population and, at the bottom (panel B) the mean difficulty of the items (black dots) and their assessment range. Panel C describes the precision of the estimate to raw score function. The solid line in panel C shows the function that relates the total raw score (from 0 to 38) to the person’s functioning level (in logits), the dotted line shows the standard error of the estimates. The items cover a large range of functioning levels, with more items in the areas of lower levels of functioning. Table 5 shows the items and testlets sorted according to their highest threshold, i.e., the location on the measurement continuum indicating that a functioning domain is first reported as a problem rather than not being a problem. The analysis of the locations of the item difficulty thresholds showed that the items that were the most likely to be reported as being a problem were the items ph046 - Hearing (mean difficulty (SD) = 1.4(2.15)), the item ph043 – Eyesight distance (1.24 (2.02)), and Testlet 7 consisting of mh002 – Sad or depressed last month and mh007 – Trouble sleeping (1.38(0.64)). The least likely to be endorsed as being a problem were the items ph049d4 - Eating, cutting up food (-2.47) and ph048d10– Picking up coins (-1.6), as well as Testlet 4 consisting of the items ph048d2 - Sitting two hours, ph049d5 - Getting in or out of bed, and ph049d6 - Using the toilet, incl getting up or down (-0.95(0.35)).

Table 6 shows the transformation table for the conversion of the observed raw total score to a psychometrically sound interval-scaled score, ranging from 0 to 100, including the precision of the person parameter estimates in terms of SEM. The corresponding graphical representation can be found in Fig 1, panel C. The solid line depicts the function that relates the total raw score (from 0 to 38) to the person’s functioning level (in logits), the dotted line shows the standard error of the estimates. The precision of the person parameter estimates decreases towards the extremes. However, the interval sizes between these estimates also increase towards the extremes so that the precision of the score-to-estimate function appears relatively steady (Fig 1, panel C). The Supporting Information makes available an R-script for the recoding of selected functioning items and the conversion of derived raw total scores to 0–100 interval-scaled scores (S1 File). Interval-scaled functioning scores are interpreted as follows: lower scores indicate lower levels of functioning (i.e., more problems) and higher scores indicate higher levels of functioning (i.e., fewer problems in functioning), where 100 stands for the highest possible functioning level that this metric can measure.

**Table 6:** Functioning score transformation table.

Ordinal-scaled raw total score	PCM logit score	SEM	Interval-scaled 0–100 score
0	‒4.25	–	0
1	‒3.41	1.04	8.8
2	‒2.64	0.75	16.8
3	‒2.19	0.61	21.5
4	‒1.87	0.52	24.9
5	‒1.63	0.47	27.4
6	‒1.43	0.43	29.5
7	‒1.26	0.40	31.2
8	‒1.11	0.37	32.8
9	‒0.98	0.35	34.2
10	‒0.86	0.34	35.4
11	‒0.75	0.33	36.6
12	‒0.64	0.32	37.7
13	‒0.54	0.32	38.8
14	‒0.44	0.31	39.8
15	‒0.35	0.31	40.8
16	‒0.25	0.31	41.8
17	‒0.16	0.31	42.7
18	‒0.06	0.31	43.8
19	0.04	0.31	44.8
20	0.14	0.32	45.9
21	0.24	0.32	46.9
22	0.35	0.33	48.1
23	0.47	0.34	49.3
24	0.59	0.35	50.6
25	0.71	0.36	51.8
26	0.85	0.37	53.3
27	1	0.39	54.9
28	1.16	0.41	56.5
29	1.33	0.43	58.3
30	1.53	0.45	60.4
31	1.75	0.48	62.7
32	2	0.52	65.3
33	2.3	0.56	68.4
34	2.65	0.61	72.1
35	3.07	0.68	76.5
36	3.61	0.79	82.1
37	4.43	1.06	90.7
38	5.32	–	100

Note: PCM = partial credit model; SEM = Standard error of measure

Finally, analysis of MIC by means of the RCI_IRT_ indicated that persons that were flagged with a significant increase or decrease in the estimated functioning parameter between two Waves (i.e., |RCI_IRT_| > 1.96) showed absolute changes of at least nine score points on the corresponding interval-scaled 0–100 functioning score.

## 4. Discussion

This study presented the results of a psychometric analysis testing the measurement properties of a newly developed functioning metric, which was built using a selection of 33 SHARE items that could be linked to functioning domains of the ICF [47]. Using a modern test theory approach, the analysis supported the validity and reliability of the functioning metric and additionally showed that the raw total score based on the retained functioning items can be converted to an interval-scaled functioning score using the transformation table provided in Table 6. The interval-scaled functioning score ranges from 0 (extreme problems in functioning) to 100 (no problems in functioning) and can also be read as a percentage. It is easy to use and is readily available for statistical analyses of health data of the ageing population in Switzerland and neighboring countries. The metric can be used to understand and model the functioning of the ageing population in Switzerland over time, complementing mortality and morbidity data. It can also be employed to model healthy ageing trajectories and to identify modifiable determinants of functioning loss, among other things. Furthermore, if sufficient data is available, the functioning metric could be employed to define population groups based on disability experience, i.e., those with no disability, those experiencing mild, moderate or severe disability.

Potential reference values for describing the importance of change in a functioning outcome are essential as they would allow valid conclusions to be drawn, for example, on the effects of potential moderators and mediators in longitudinal modeling of the functioning scores. Moreover, such reference values for meaningful change would support the calculation of sample sizes and power analyses in data-driven health-related research. In this study, we used the so-called IRT-based reliable change index, the RCIIRT [42] with absolute values above 1.96 indicating reliable significant differences. However, using the statistical significance to discuss the importance of change entails some risks. A statistically significant change may or may not be significant in the perception of the individual, and vice versa, i.e., participants, researchers, health professionals, and society may perceive the amount of change necessary to be important differently [48]. Effective approaches to determine if a change has a meaningful impact on the person’s life include, for example, anchor-based approaches. Such approaches link an outcome measure to an objective measure of perceived change in order to compute cut-off points.

Our study is comparable to previous studies, such as for example the ‘Ageing Trajectories of Health: Longitudinal Opportunities and Synergies’ study or ATHLOS [49], the ‘Model Disability Survey’ or MDS [15], and the study on ‘Psychosocial Factors Relevant to Brain Disorders in Europe’ or PARADISE [10], that developed and tested metrics of functioning using modern test theory models. All three studies used an ICF-based approach for the selection of items to assess relevant domains of functioning. Other similarities include the content and alignment of functioning items with regard to their difficulty (i.e., how easy or demanding an activity or task is according to the number of respondents who reported it as a problem). For example, items assessing functioning with regard to eating, picking up smaller objects, making phone calls, and using the toilet were only reported as a problem by people with very low levels of functioning. In contrast, items that assess emotional functions and energy level were commonly observed as demanding, even for people with high levels of functioning. However, we also observed some notable discrepancies between our findings and the aforementioned studies. For example, contrary to the MDS and ATHLOS, the item assessing hearing loss was easily reported as a problem in our analysis using SHARE data. This discrepancy can be explained with the characteristics of the populations: while the focus of SHARE is persons 50 years of age or older, ATHLOS focused on persons older than 40 years of age, and the MDS included the adult population irrespective of age. Importantly, the prevalence of hearing loss is reported to be 5% in adults 45–54 years of age, while 55% in adults 75 and older [50].

Also, differences in the operationalization of functioning domains can drastically influence the prevalence of a functioning problem in a population and, thus, the difficulty of an item. This is observed, for example, with items assessing pain. In ATHLOS, the item asks if the participants experience some degree of pain or not, and is so easily endorsed as problematic. Whereas in the MDS, problems in day-to-day life due to pain can be rated from 1 (no problem) to 5 (extreme problem), taking into account the effect of medication. Since participants are able to indicate that pain does not lead to problems in daily life, it can be expected that more participants would report no or fewer problems because of pain in the MDS versus in ATHLOS. In our study, pain was not taken into account for the development of the functioning metric, due to the different assessment strategies across SHARE Waves and a high amount of missing values.

The population targeting of any metric, including the one in this study, is very important because it gives an indication of its suitability for a given population. The metric should neither be too easy nor too difficult to assess the full range of functioning levels. Specifically, targeting describes how well the difficulties of assessment items match a population’s ability levels [33]. When developing a metric, the selection of representative and well-operationalized items that can cover the largest possible range of the functioning continuum of a target population is essential. In this study, the selection of items to construct the functioning metric depended entirely on information already available in SHARE. Given a previous detailed analysis of how functioning is collected in Swiss data sources, we confirm that the developed metric covers important domains of functioning. We observed, however, that on average, selected functioning items from SHARE tend to assess higher levels of problems than what is observed in the study population. In other words, we found a discrepancy between the range of the functioning continuum that is covered by the selected functioning items and the actual functioning level of the population. The potential reason for this is twofold. First, it is known that the survey population contains a higher proportion of persons from lower age groups, i.e., 50–75 years. We also argue that persons with lower levels of functioning or persons who moved to assisted living or were hospitalized are more likely to drop out of the survey or to refuse participation [22]. Second, the information collected by the metric may be compromised by the dichotomous rating of many items. The dichotomous ratings do not inform on the extent of the difficulties, which can be mild to extreme. In this regard, items with ordinal response categories, with middle categories, instead of dichotomously rated items, could support a more refined assessment of the severity of a functioning problem. Despite these concerns, we showed that the 33 items used in this metric are sufficient to provide a broad measurement scope for a robust assessment of functioning, and are appropriate for modeling functioning and studying its determinants in older populations living in Switzerland and neighbouring countries.

Our study has limitations. Since the analysis was based on a selected set of items rather than on a complete questionnaire, some issues had to be addressed to ensure a sound metric. First, the initial analysis revealed some local item dependencies and multidimensionality. This issue was solved by aggregating items into testlets. This is a common approach, and testlets have been used in other studies to solve similar problems [51, 52]. Second, the item assessing eyesight in reading (with glasses or lenses) was found insufficient in discriminating between different levels of functioning. In fact, poor eyesight in reading is a widespread problem among older adults, thus, aligning this item on a functioning metric is challenging, since glasses can further provide effective correction at all levels. Third, DIF was found for age, i.e., with increasing age, the perceived difficulty of the items changed. Age and functioning are on a causal pathway, with more functioning problems with increasing age. The DIF for age is understood as supporting the effect of the age of the respondent on the reported difficulty with functioning items, rather than evidence of bias [53, 54]. We expect that resolving the DIF, e.g., by providing age-specific item difficulty estimates, will decrease the validity of the metric [55]. Age-adjusted item parameters may level the person parameter differences. To keep the functioning scores comparable across age groups, we decided to only report the age DIF and not to adjust for it. The absence of DIF for the assessment Waves supported the stability of selected items over time and no DIF was found for the countries.

## 5. Conclusion

This study confirmed the validity and reliability of a newly developed functioning metric based on selected SHARE items. The transformation table can be used to derive a functioning score based on the observed responses of an individual to these items and their corresponding raw total score. The resulting interval-scaled functioning score ranging from 0 to 100, i.e., from extreme to no problems in functioning, is easy to use, can be read as a percentage, and fosters an intuitive understanding of a person’s or population’s functioning level. The developed functioning metric and corresponding interval-scaled score is readily available for statistical analyses of health data in Switzerland and neighboring countries as well as for aggregation into a summary statistic, e.g., mean or median, to be used as an indicator of the functioning level of an ageing population.

## Supporting information

S1 File R-syntax for Item Recoding and Score Transformation (DOCX)

S2 File Overview of Response Frequencies and Missing Values for the Total Sample and per Country. (DOCX)

S1 Fig Local Item Dependencies at Start. (DOCX)
