# Supplementary material for: Development of a functioning metric for the ageing population using data from the survey of health, ageing and retirement in Europe (SHARE)
Source: PLoS One. 2025 Apr 24;20(4):e0320068. doi: 10.1371/journal.pone.0320068 (PMC12021154; doi:10.1371/journal.pone.0320068)
Supplement: S1 File — (DOCX) [file pone.0320068.s001.docx]

# S1 File. R-syntax for Item Recoding and Score Transformation.

#*******************************************************************

#

#Syntax for the preparation of the functioning items and obtaining

#the Rasch-based 0-100 score.

#In this study, the data ("Share_dta") consisted of data

#from the waves 1,2,4,5,6,7,8

#

#*******************************************************************

#Variable selection-

#Following variables were retained for the functioning metric.

functioning_variables = data.frame(matrix(c(

"ph005_", "Limited activities because of health",

"ph043_", "Eyesight distance",

"ph044_", "Eyesight reading",

"ph046_", "Hearing",

"ph048d1", "Difficulties walking 100 meter",

"ph048d2", "Difficulties sitting two hours",

"ph048d3", "Difficulties getting up from chair",

"ph048d4", "Difficulties climbing several flights of stairs",

"ph048d5", "Difficulties climbing one flight of stairs",

"ph048d6", "Difficulties stooping, kneeling, crouching",

"ph048d7", "Difficulties reaching or extending arms above shoulder",

"ph048d8", "Difficulties pulling or pushing large objects",

"ph048d9", "Difficulties lifting or carrying weights over 5 kilos",

"ph048d10", "Difficulties picking up a small coin from a table",

"ph049d1", "Difficulties dressing, including shoes and socks",

"ph049d2", "Difficulties walking across a room",

"ph049d3", "Difficulties bathing or showering",

"ph049d4", "Difficulties eating, cutting up food",

"ph049d5", "Difficulties getting in or out of bed",

"ph049d6", "Difficulties using the toilet, incl getting up or",

"ph049d7", "Difficulties using a map in a strange place",

"ph049d8", "Difficulties preparing a hot meal",

"ph049d9", "Difficulties shopping for groceries",

"ph049d10", "Difficulties telephone calls",

"ph049d11", "Difficulties taking medications",

"ph049d12", "Difficulties doing work around the house or garden",

"ph049d13", "Difficulties managing money",

"mh002_", "Feeling sad",

"mh007_", "Trouble sleeping",

"mh010_", "Irritability",

"mh013_", "Fatigue",

"mh014_", "Difficulty concentrate",

"mh015_", "Concentrating on reading"), ncol = 2, byrow = TRUE))

#**********************************************************************

#Set to missing-

#set all the -2 (Refusal) and -1 (Don't know) to NA, -3 implausible

#to missing

all_variables = functioning_variables[,1]

for(i in 1:length(all_variables)){

SHARE_dta[which(SHARE_dta[, all_variables[i] ] == -3), all_variables[i]] = NA

SHARE_dta[which(SHARE_dta[, all_variables[i] ] == -2), all_variables[i]] = NA

SHARE_dta[which(SHARE_dta[, all_variables[i] ] == -1), all_variables[i]] = NA

}

#**************************************************

#Recode variables-

#*Feeling sad in the last month "mh002_"

#*1 = yes

SHARE_dta[ which(SHARE_dta[, "mh002_" ] == 5), "mh002_" ] = 0

#**************************************************

#*Trouble sleeping the last month "mh007_"

#* 1 = Trouble with sleep

SHARE_dta[ which(SHARE_dta[, "mh007_" ] == 5), "mh007_" ] = 0

SHARE_dta[ which(SHARE_dta[, "mh007_" ] == 2), "mh007_" ] = 0

#**************************************************

#*Irritability the last month "mh010_"

#* 1 = yes

SHARE_dta[ which(SHARE_dta[, "mh010_" ] == 5), "mh010_" ] = 0

#**************************************************

#*Fatigue "mh013_" 1 = yes

SHARE_dta[ which(SHARE_dta[, "mh013_" ] == 5), "mh013_" ] = 0

#***************************************************

#"mh014_" 1 = difficulty in concentrating 5 = no difficulty#

#code 2 found in some waves means no such difficulty

SHARE_dta[which(SHARE_dta[, "mh014_"] == 5), "mh014_"] = 0

SHARE_dta[which(SHARE_dta[, "mh014_"] == 2), "mh014_"] = 0

#****************************************************

#"mh015_" Concentrating on reading, 1 = difficulty 5 = no difficulty

#code 2 found in some waves means no such difficulty

SHARE_dta[which(SHARE_dta[, "mh015_"] == 5), "mh015_"] = 0

SHARE_dta[which(SHARE_dta[, "mh015_"] == 2), "mh015_"] = 0

#****************************************************

#*"ph005_" Limited in activities because of health 1 = severely limited 4 not limited

#* reversed coding

SHARE_dta[, "ph005_"] = abs((SHARE_dta[, "ph005_"])-4)

#*****************************************************

#* "ph043_" Eyesight distance

SHARE_dta[which(SHARE_dta[, "ph043_"] == 6), "ph043_"] = NA

#******************************************************

#* "ph044_" Eyesight reading

SHARE_dta[which(SHARE_dta[, "ph044_"] == 6), "ph044_"] = NA

#******************************************************

#*"ph046_" Hearing - no change

#******************************************************

#*"ph048d1" difficulties walking 100 meter - no change

#****************************************************

#*"ph048d2" Difficulties: sitting two hours - no change

#*#***************************************************

#* "ph048d3" Difficulties: getting up from chair - no change

#*#***************************************************

#* "ph048d4" Difficulties: climbing several flights of stairs - no change

#*#***************************************************

#*"ph048d5" Difficulties: climbing one flight of stairs - no change

#***************************************************

#*"ph048d6" Difficulties: stooping, kneeling, crouching - no change

#*#***************************************************

#* "ph048d7" Difficulties: reaching or extending arms above shoulder - no change

#*#***************************************************

#*"ph048d8" Difficulties: pulling or pushing large objects - no change

#*#***************************************************

#* "ph048d9" Difficulties: lifting or carrying weights over 5 kilos - no change

#*#***************************************************

#*"ph048d10" Difficulties: picking up a small coin from a table - no change

#*#***************************************************

#*"ph049d1" Difficulties: dressing, including shoes and socks - no change

#*#**************************************************

#* "ph049d2" Difficulties: walking across a room - no change

#*#***************************************************

#* "ph049d3" Difficulties: bathing or showering - no change

#***************************************************

#*"ph049d4" Difficulties: eating, cutting up food - no change

#***************************************************

#*"ph049d5" Difficulties: getting in or out of bed - no change

#****************************************************

#*"ph049d6" Difficulties: using the toilet, incl getting up or - no change

#***************************************************

#*"ph049d7" Difficulties: using a map in a strange place - no change

#************************************************

#*"ph049d8" Difficulties: preparing a hot meal - no change

#****************************************************

#*"ph049d9" Difficulties: shopping for groceries - no change

#****************************************************

#*"ph049d10" Difficulties: telephone calls - no change

#****************************************************

#*"ph049d11" Difficulties: taking medications - no change

#****************************************************

#*"ph049d12" Difficulties: doing work around the house or garden - no change

#****************************************************

#*"ph049d13" Difficulties: managing money - no change

#***************************************************

#set minimum to zero for the variables where the minimum is at 1

Min_per_var = apply(SHARE_dta[, all_variables],2,min, na.rm = TRUE)

SHARE_dta[, names(which(Min_per_var == 1))] = SHARE_dta[, names(which(Min_per_var == 1))] - 1

#reverse code the variables: higher score = better functioning

rev_code = function(x){

x = abs(x - max(x, na.rm = TRUE))

return(x)

}

SHARE_dta[, all_variables] = apply(data.All[, all_variables], 2, rev_code)

#Transformation table--------------------------------

TT = as.data.frame(matrix(c(

0, -4.25, 0,

1, -3.41, 8.8,

2, -2.64, 16.8,

3, -2.19, 21.5,

4, -1.87, 24.9,

5, -1.63, 27.4,

6, -1.43, 29.5,

7, -1.26, 31.2,

8, -1.11, 32.8,

9, -0.98, 34.2,

10, -0.86, 35.4,

11, -0.75, 36.6,

12, -0.64, 37.7,

13, -0.54, 38.8,

14, -0.44, 39.8,

15, -0.35, 40.8,

16, -0.25, 41.8,

17, -0.16, 42.7,

18, -0.06, 43.8,

19, 0.04, 44.8,

20, 0.14, 45.9,

21, 0.24, 46.9,

22, 0.35, 48.1,

23, 0.47, 49.3,

24, 0.59, 50.6,

25, 0.71, 51.8,

26, 0.85, 53.3,

27, 1, 54.9,

28, 1.16, 56.5,

29, 1.33, 58.3,

30, 1.53, 60.4,

31, 1.75, 62.7,

32, 2, 65.3,

33, 2.3, 68.4,

34, 2.65, 72.1,

35, 3.07, 76.5,

36, 3.61, 82.1,

37, 4.43, 90.7,

38, 5.32, 100), ncol = 3, byrow = TRUE))

colnames(TT) = c("Raw_Score", "Logit_PP", "Score_0_100")

#********************************************************************

#Score Transformation-

#works only for complete case data

#missing values would need to be imputed

SHARE_dta[, "raw_scores"] = rowSums(SHARE_dta[, functioning_variables[,1]])

#new columns in the data: raw score control variable (for verification),the person parameter in logits and the 0-100 score,

SHARE_dta[i, c("raw_score_check", "person_param_logit", "score_0_100")] = NA

#adding the person_parameter and 0-100 score to the dataset, row by row.

for(i in 1:nrow(SHARE_dta)){

SHARE_dta[i, c("raw_score_check", "person_param_logit", "score_0_100")] =

TT[which(TT[, "Raw_score"]%in%SHARE_dta[i, "raw_scores"]),

c("Raw_score", "Logit_PP", "Score_0_100")]

}
