# Supplementary material for: Development of a functioning metric for the ageing population using data from the survey of health, ageing and retirement in Europe (SHARE)
Source: PLoS One. 2025 Apr 24;20(4):e0320068. doi: 10.1371/journal.pone.0320068 (PMC12021154; doi:10.1371/journal.pone.0320068)
Supplement: S2 File — (DOCX) [file pone.0320068.s002.docx]

# S2 File. Overview of Response Frequencies and Missing Values for the Total Sample and per Country

**Table 1.** Response frequencies across all included countries (N = 34092).

**Table 2.** Response Frequencies for SHARE Switzerland after imputation (N = 4350).

**Fig 1.** Overview of missing observations in SHARE Switzerland.

**Table 3.** Response Frequencies for SHARE France after imputation (N = 7526).

**Fig 2.** Overview of missing observations in SHARE France.

**Table 4.** Response Frequencies for SHARE Austria after imputation (N = 5874).

**Fig 3.** Overview of missing observations in SHARE Austria.

**Table 5.** Response Frequencies for SHARE Germany after imputation (N = 8279).

**Fig 4.** Overview of missing observations in SHARE Germany.

**Table 6.** Response Frequencies for SHARE Italy after imputation (N = 8063).

**Fig 5.** Overview of missing observations in SHARE Italy.

**Table 1. Response frequencies across all included countries (N = 34092).**

| **Item** | **Response options** | | | | |
| --- | --- | --- | --- | --- | --- |
|  | 1=Severely limited,  n (%) | 2=Limited, but not severely, n (%) | 3=Not limited, n (%) |  |  |
| ph005 | 18663 (54.74%) | 10111 (29.66%) | 5318 (15.6%) |  |  |
|  | 1=Excellent, n (%) | 2=Very good, n (%) | 3=Good, n (%) | 4=Fair, n (%) | 5=Poor, n (%) |
| ph043 | 5096 (14.95%) | 10748 (31.53%) | 13913 (40.81%) | 3194 (9.37%) | 1141 (3.35%) |
| ph044 | 4193 (12.3%) | 10084 (29.58%) | 13260 (38.89%) | 4251 (12.47%) | 2304 (6.76%) |
| ph046 | 4076 (11.96%) | 9786 (28.7%) | 14174 (41.58%) | 4940 (14.49%) | 1116 (3.27%) |
|  | Not selected, n (%) | 1=Selected, n (%) |  |  |  |
| ph048d1 | 30414 (89.21%) | 3678 (10.79%) |  |  |  |
| ph048d2 | 30637 (89.87%) | 3455 (10.13%) |  |  |  |
| ph048d3 | 27982 (82.08%) | 6110 (17.92%) |  |  |  |
| ph048d4 | 24908 (73.06%) | 9184 (26.94%) |  |  |  |
| ph048d5 | 29934 (87.8%) | 4158 (12.2%) |  |  |  |
| ph048d6 | 23928 (70.19%) | 10164 (29.81%) |  |  |  |
| ph048d7 | 30548 (89.6%) | 3544 (10.4%) |  |  |  |
| ph048d8 | 29544 (86.66%) | 4548 (13.34%) |  |  |  |
| ph048d9 | 27197 (79.78%) | 6895 (20.22%) |  |  |  |
| ph048d10 | 32450 (95.18%) | 1642 (4.82%) |  |  |  |
| ph049d1 | 31396 (92.09%) | 2696 (7.91%) |  |  |  |
| ph049d2 | 33248 (97.52%) | 844 (2.48%) |  |  |  |
| ph049d3 | 31932 (93.66%) | 2160 (6.34%) |  |  |  |
| ph049d4 | 33312 (97.71%) | 780 (2.29%) |  |  |  |
| ph049d5 | 32861 (96.39%) | 1231 (3.61%) |  |  |  |
| ph049d6 | 33164 (97.28%) | 928 (2.72%) |  |  |  |
| ph049d7 | 31461 (92.28%) | 2631 (7.72%) |  |  |  |
| ph049d8 | 32708 (95.94%) | 1384 (4.06%) |  |  |  |
| ph049d9 | 31719 (93.04%) | 2373 (6.96%) |  |  |  |
| ph049d10 | 33313 (97.72%) | 779 (2.28%) |  |  |  |
| ph049d11 | 33261 (97.56%) | 831 (2.44%) |  |  |  |
| ph049d12 | 30254 (88.74%) | 3838 (11.26%) |  |  |  |
| ph049d13 | 32628 (95.71%) | 1464 (4.29%) |  |  |  |
|  | 1=Yes, n (%) | 5=No, n (%) |  |  |  |
| mh002 | 20394 (59.82%) | 13698 (40.18%) |  |  |  |
| mh007 | 23175 (67.98%) | 10917 (32.02%) |  |  |  |
| mh010 | 24589 (72.13%) | 9503 (27.87%) |  |  |  |
| mh013 | 22823 (66.95%) | 11269 (33.05%) |  |  |  |
| mh014 | 29707 (87.14%) | 4385 (12.86%) |  |  |  |
| mh015 | 29307 (85.96%) | 4785 (14.04%) |  |  |  |

**Table 2. Response Frequencies for SHARE Switzerland after imputation (N = 4350).**

| **Item** | **Response Options** |  |  |  |  |
| --- | --- | --- | --- | --- | --- |
|  | 1=Severely limited,  n (%) | 2=Limited, but not severely, n (%) | 3=Not limited, n (%) |  |  |
| ph005 | 2828 (65.01%) | 1113 (25.59%) | 409 (9.4%) |  |  |
|  | 1=Excellent, n (%) | 2=Very good, n (%) | 3=Good, n (%) | 4=Fair, n (%) | 5=Poor, n (%) |
| ph043 | 747 (17.17%) | 1733 (39.84%) | 1587 (36.48%) | 217 (4.99%) | 66 (1.52%) |
| ph044 | 595 (13.68%) | 1585 (36.44%) | 1573 (36.16%) | 382 (8.78%) | 215 (4.94%) |
| ph046 | 476 (10.94%) | 1427 (32.8%) | 1921 (44.16%) | 481 (11.06%) | 45 (1.03%) |
|  | Not selected, n (%) | 1=Selected, n (%) |  |  |  |
| ph048d1 | 4098 (94.21%) | 252 (5.79%) |  |  |  |
| ph048d2 | 4059 (93.31%) | 291 (6.69%) |  |  |  |
| ph048d3 | 3806 (87.49%) | 544 (12.51%) |  |  |  |
| ph048d4 | 3786 (87.03%) | 564 (12.97%) |  |  |  |
| ph048d5 | 4149 (95.38%) | 201 (4.62%) |  |  |  |
| ph048d6 | 3469 (79.75%) | 881 (20.25%) |  |  |  |
| ph048d7 | 4135 (95.06%) | 215 (4.94%) |  |  |  |
| ph048d8 | 4102 (94.3%) | 248 (5.7%) |  |  |  |
| ph048d9 | 3828 (88%) | 522 (12%) |  |  |  |
| ph048d10 | 4208 (96.74%) | 142 (3.26%) |  |  |  |
| ph049d1 | 4159 (95.61%) | 191 (4.39%) |  |  |  |
| ph049d2 | 4322 (99.36%) | 28 (0.64%) |  |  |  |
| ph049d3 | 4211 (96.8%) | 139 (3.2%) |  |  |  |
| ph049d4 | 4310 (99.08%) | 40 (0.92%) |  |  |  |
| ph049d5 | 4290 (98.62%) | 60 (1.38%) |  |  |  |
| ph049d6 | 4310 (99.08%) | 40 (0.92%) |  |  |  |
| ph049d7 | 4164 (95.72%) | 186 (4.28%) |  |  |  |
| ph049d8 | 4273 (98.23%) | 77 (1.77%) |  |  |  |
| ph049d9 | 4208 (96.74%) | 142 (3.26%) |  |  |  |
| ph049d10 | 4308 (99.03%) | 42 (0.97%) |  |  |  |
| ph049d11 | 4320 (99.31%) | 30 (0.69%) |  |  |  |
| ph049d12 | 4060 (93.33%) | 290 (6.67%) |  |  |  |
| ph049d13 | 4253 (97.77%) | 97 (2.23%) |  |  |  |
|  | 1=Yes, n (%) | 5=No, n (%) |  |  |  |
| mh002 | 2709 (62.28%) | 1641 (37.72%) |  |  |  |
| mh007 | 3144 (72.28%) | 1206 (27.72%) |  |  |  |
| mh010 | 3282 (75.45%) | 1068 (24.55%) |  |  |  |
| mh013 | 3150 (72.41%) | 1200 (27.59%) |  |  |  |
| mh014 | 3998 (91.91%) | 352 (8.09%) |  |  |  |
| mh015 | 4011 (92.21%) | 339 (7.79%) |  |  |  |


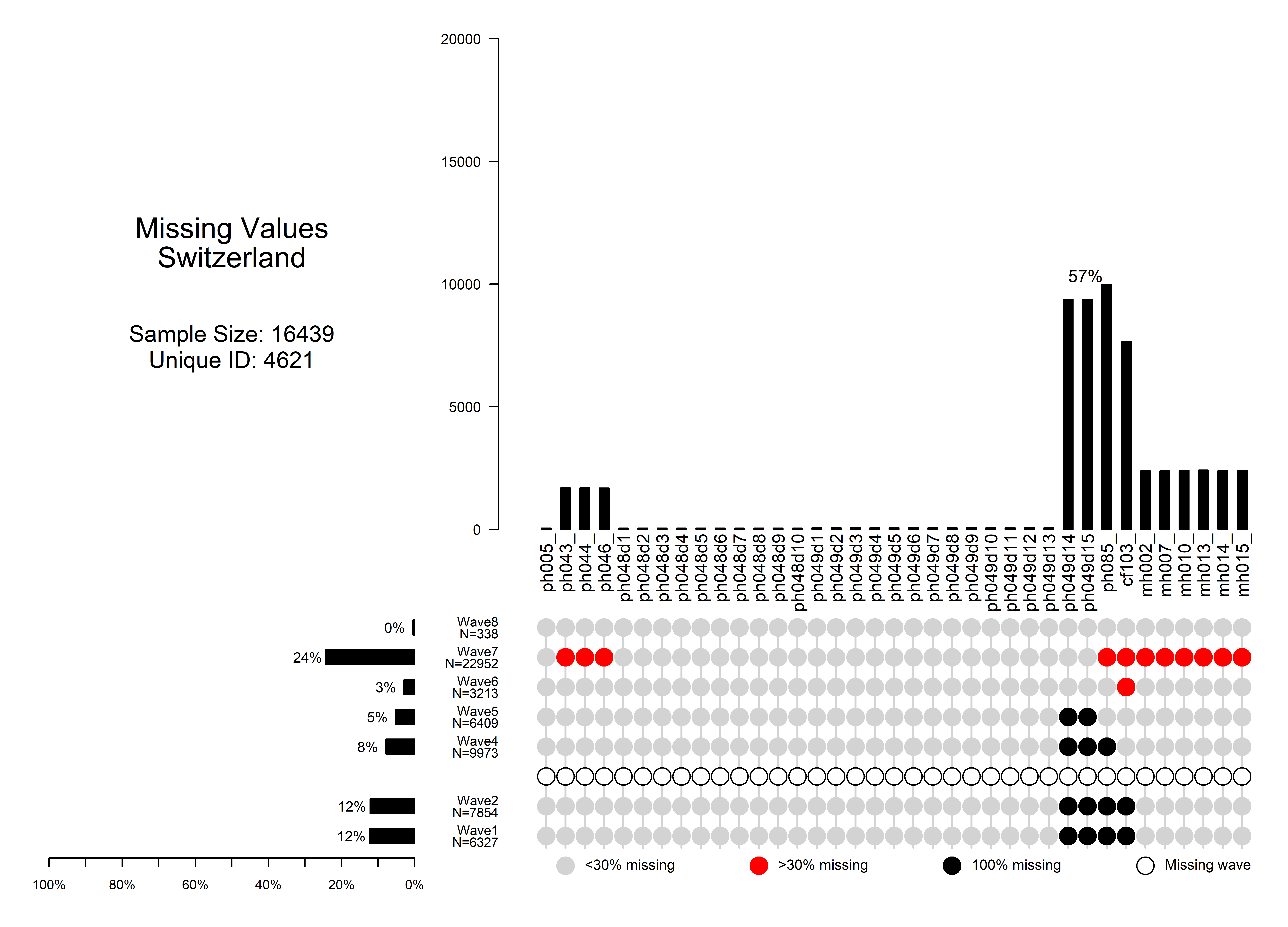


**Fig 1. Overview of missing observations in SHARE Switzerland.**

**Table 3. Response Frequencies for SHARE France after imputation (N = 7526).**

| **Item** | **Response options** |  |  |  |  |
| --- | --- | --- | --- | --- | --- |
|  | 1=Severely limited,  n (%) | 2=Limited, but not severely, n (%) | 3=Not limited, n (%) |  |  |
| ph005 | 4170 (55.41%) | 2077 (27.6%) | 1279 (16.99%) |  |  |
|  | 1=Excellent, n (%) | 2=Very good, n (%) | 3=Good, n (%) | 4=Fair, n (%) | 5=Poor, n (%) |
| ph043 | 1601 (21.27%) | 2088 (27.74%) | 2796 (37.15%) | 721 (9.58%) | 320 (4.25%) |
| ph044 | 1385 (18.4%) | 2035 (27.04%) | 2699 (35.86%) | 824 (10.95%) | 583 (7.75%) |
| ph046 | 952 (12.65%) | 1773 (23.56%) | 3103 (41.23%) | 1298 (17.25%) | 400 (5.31%) |
|  | Not selected, n (%) | 1=Selected, n (%) |  |  |  |
| ph048d1 | 6636 (88.17%) | 890 (11.83%) |  |  |  |
| ph048d2 | 6779 (90.07%) | 747 (9.93%) |  |  |  |
| ph048d3 | 6262 (83.2%) | 1264 (16.8%) |  |  |  |
| ph048d4 | 5291 (70.3%) | 2235 (29.7%) |  |  |  |
| ph048d5 | 6627 (88.05%) | 899 (11.95%) |  |  |  |
| ph048d6 | 5184 (68.88%) | 2342 (31.12%) |  |  |  |
| ph048d7 | 6620 (87.96%) | 906 (12.04%) |  |  |  |
| ph048d8 | 6325 (84.04%) | 1201 (15.96%) |  |  |  |
| ph048d9 | 5862 (77.89%) | 1664 (22.11%) |  |  |  |
| ph048d10 | 7208 (95.77%) | 318 (4.23%) |  |  |  |
| ph049d1 | 6768 (89.93%) | 758 (10.07%) |  |  |  |
| ph049d2 | 7337 (97.49%) | 189 (2.51%) |  |  |  |
| ph049d3 | 7012 (93.17%) | 514 (6.83%) |  |  |  |
| ph049d4 | 7378 (98.03%) | 148 (1.97%) |  |  |  |
| ph049d5 | 7262 (96.49%) | 264 (3.51%) |  |  |  |
| ph049d6 | 7338 (97.5%) | 188 (2.5%) |  |  |  |
| ph049d7 | 6985 (92.81%) | 541 (7.19%) |  |  |  |
| ph049d8 | 7238 (96.17%) | 288 (3.83%) |  |  |  |
| ph049d9 | 6896 (91.63%) | 630 (8.37%) |  |  |  |
| ph049d10 | 7330 (97.4%) | 196 (2.6%) |  |  |  |
| ph049d11 | 7349 (97.65%) | 177 (2.35%) |  |  |  |
| ph049d12 | 6552 (87.06%) | 974 (12.94%) |  |  |  |
| ph049d13 | 7167 (95.23%) | 359 (4.77%) |  |  |  |
|  | 1=Yes, n (%) | 5=No, n (%) |  |  |  |
| mh002 | 3961 (52.63%) | 3565 (47.37%) |  |  |  |
| mh007 | 4774 (63.43%) | 2752 (36.57%) |  |  |  |
| mh010 | 5094 (67.69%) | 2432 (32.31%) |  |  |  |
| mh013 | 4574 (60.78%) | 2952 (39.22%) |  |  |  |
| mh014 | 6505 (86.43%) | 1021 (13.57%) |  |  |  |
| mh015 | 6325 (84.04%) | 1201 (15.96%) |  |  |  |


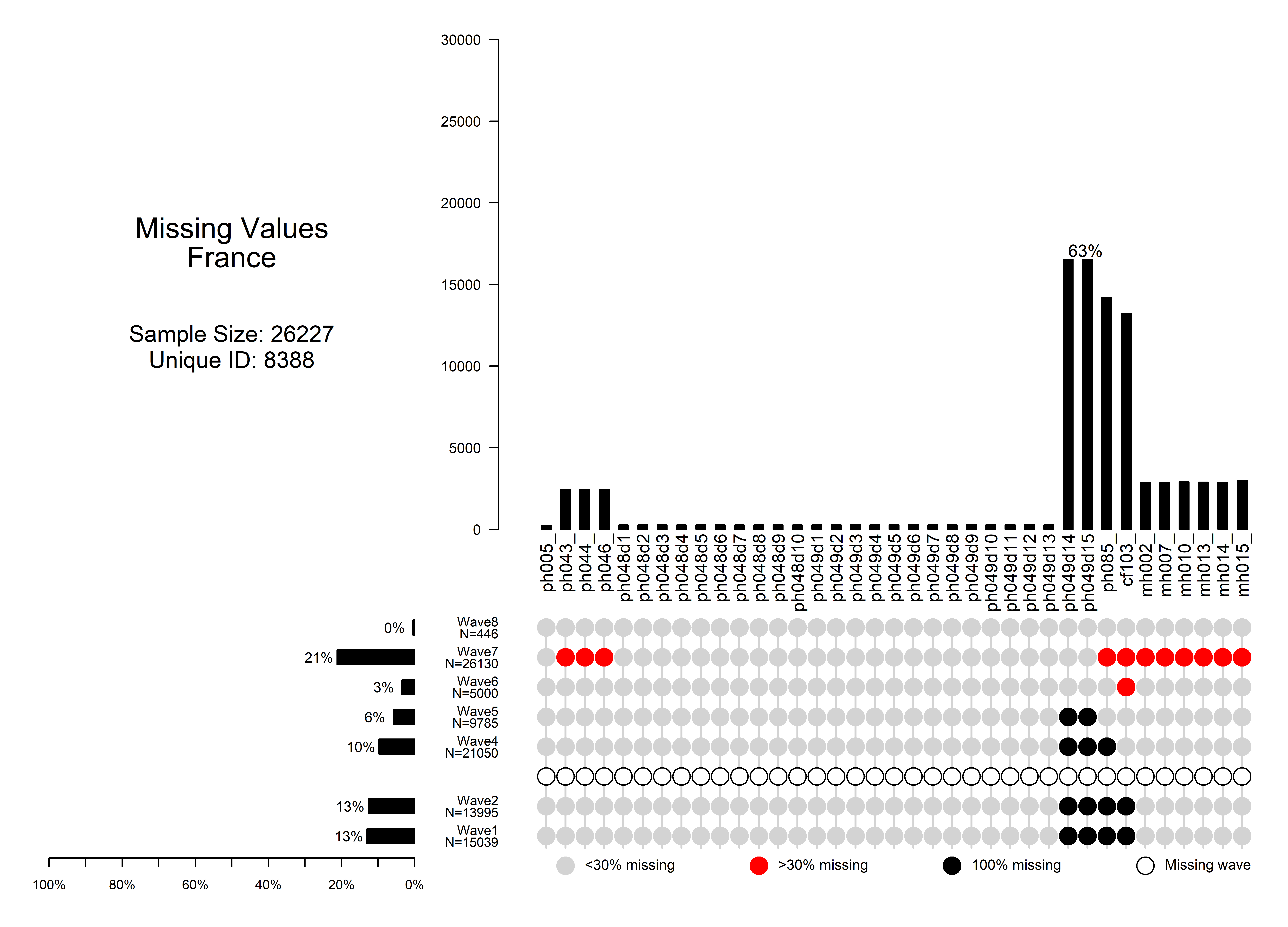


**Fig 2. Overview of missing observations in SHARE France.**

**Table 4. Response Frequencies for SHARE Austria after imputation (N = 5874).**

| **Item** | **Response options** |  |  |  |  |
| --- | --- | --- | --- | --- | --- |
|  | 1=Severely limited,  n (%) | 2=Limited, but not severely, n (%) | 3=Not limited, n (%) |  |  |
| ph005 | 2959 (50.37%) | 1975 (33.62%) | 940 (16%) |  |  |
|  | 1=Excellent, n (%) | 2=Very good, n (%) | 3=Good, n (%) | 4=Fair, n (%) | 5=Poor, n (%) |
| ph043 | 776 (13.21%) | 2609 (44.42%) | 1991 (33.9%) | 373 (6.35%) | 125 (2.13%) |
| ph044 | 670 (11.41%) | 2446 (41.64%) | 1773 (30.18%) | 611 (10.4%) | 374 (6.37%) |
| ph046 | 832 (14.16%) | 2221 (37.81%) | 2013 (34.27%) | 664 (11.3%) | 144 (2.45%) |
|  | Not selected, n (%) | 1=Selected, n (%) |  |  |  |
| ph048d1 | 5284 (89.96%) | 590 (10.04%) |  |  |  |
| ph048d2 | 5276 (89.82%) | 598 (10.18%) |  |  |  |
| ph048d3 | 4729 (80.51%) | 1145 (19.49%) |  |  |  |
| ph048d4 | 3935 (66.99%) | 1939 (33.01%) |  |  |  |
| ph048d5 | 5033 (85.68%) | 841 (14.32%) |  |  |  |
| ph048d6 | 4139 (70.46%) | 1735 (29.54%) |  |  |  |
| ph048d7 | 5200 (88.53%) | 674 (11.47%) |  |  |  |
| ph048d8 | 5030 (85.63%) | 844 (14.37%) |  |  |  |
| ph048d9 | 4577 (77.92%) | 1297 (22.08%) |  |  |  |
| ph048d10 | 5469 (93.11%) | 405 (6.89%) |  |  |  |
| ph049d1 | 5436 (92.54%) | 438 (7.46%) |  |  |  |
| ph049d2 | 5731 (97.57%) | 143 (2.43%) |  |  |  |
| ph049d3 | 5513 (93.85%) | 361 (6.15%) |  |  |  |
| ph049d4 | 5727 (97.5%) | 147 (2.5%) |  |  |  |
| ph049d5 | 5650 (96.19%) | 224 (3.81%) |  |  |  |
| ph049d6 | 5710 (97.21%) | 164 (2.79%) |  |  |  |
| ph049d7 | 5268 (89.68%) | 606 (10.32%) |  |  |  |
| ph049d8 | 5579 (94.98%) | 295 (5.02%) |  |  |  |
| ph049d9 | 5433 (92.49%) | 441 (7.51%) |  |  |  |
| ph049d10 | 5761 (98.08%) | 113 (1.92%) |  |  |  |
| ph049d11 | 5750 (97.89%) | 124 (2.11%) |  |  |  |
| ph049d12 | 5002 (85.15%) | 872 (14.85%) |  |  |  |
| ph049d13 | 5566 (94.76%) | 308 (5.24%) |  |  |  |
|  | 1=Yes, n (%) | 5=No, n (%) |  |  |  |
| mh002 | 3871 (65.9%) | 2003 (34.1%) |  |  |  |
| mh007 | 4120 (70.14%) | 1754 (29.86%) |  |  |  |
| mh010 | 4879 (83.06%) | 995 (16.94%) |  |  |  |
| mh013 | 4166 (70.92%) | 1708 (29.08%) |  |  |  |
| mh014 | 5217 (88.82%) | 657 (11.18%) |  |  |  |
| mh015 | 5276 (89.82%) | 598 (10.18%) |  |  |  |


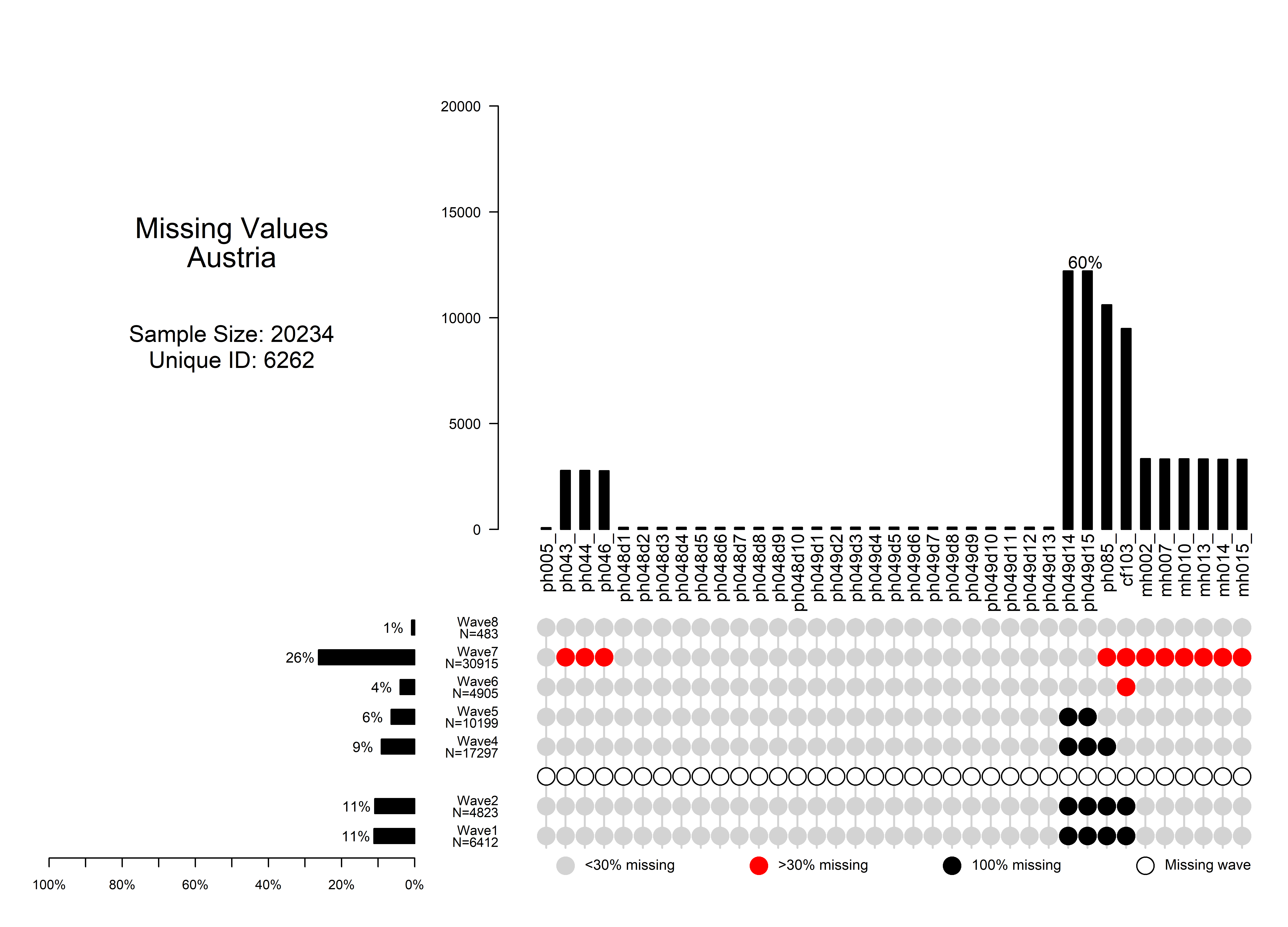


**Fig 3. Overview of missing observations in SHARE Austria.**

**Table 5. Response Frequencies for SHARE Germany after imputation (N = 8279).**

| **Item** | **Response options** |  |  |  |  |
| --- | --- | --- | --- | --- | --- |
|  | 1=Severely limited,  n (%) | 2=Limited, but not severely, n (%) | 3=Not limited, n (%) |  |  |
| ph005 | 3923 (47.38%) | 2842 (34.33%) | 1514 (18.29%) |  |  |
|  | 1=Excellent, n (%) | 2=Very good, n (%) | 3=Good, n (%) | 4=Fair, n (%) | 5=Poor, n (%) |
| ph043 | 1262 (15.24%) | 2993 (36.15%) | 3309 (39.97%) | 499 (6.03%) | 216 (2.61%) |
| ph044 | 1040 (12.56%) | 2882 (34.81%) | 3118 (37.66%) | 752 (9.08%) | 487 (5.88%) |
| ph046 | 883 (10.67%) | 2321 (28.03%) | 3640 (43.97%) | 1247 (15.06%) | 188 (2.27%) |
|  | Not selected, n (%) | 1=Selected, n (%) |  |  |  |
| ph048d1 | 7408 (89.48%) | 871 (10.52%) |  |  |  |
| ph048d2 | 7314 (88.34%) | 965 (11.66%) |  |  |  |
| ph048d3 | 6550 (79.12%) | 1729 (20.88%) |  |  |  |
| ph048d4 | 6404 (77.35%) | 1875 (22.65%) |  |  |  |
| ph048d5 | 7518 (90.81%) | 761 (9.19%) |  |  |  |
| ph048d6 | 5527 (66.76%) | 2752 (33.24%) |  |  |  |
| ph048d7 | 7422 (89.65%) | 857 (10.35%) |  |  |  |
| ph048d8 | 7186 (86.8%) | 1093 (13.2%) |  |  |  |
| ph048d9 | 6862 (82.88%) | 1417 (17.12%) |  |  |  |
| ph048d10 | 7862 (94.96%) | 417 (5.04%) |  |  |  |
| ph049d1 | 7642 (92.31%) | 637 (7.69%) |  |  |  |
| ph049d2 | 8098 (97.81%) | 181 (2.19%) |  |  |  |
| ph049d3 | 7778 (93.95%) | 501 (6.05%) |  |  |  |
| ph049d4 | 8075 (97.54%) | 204 (2.46%) |  |  |  |
| ph049d5 | 7978 (96.36%) | 301 (3.64%) |  |  |  |
| ph049d6 | 8051 (97.25%) | 228 (2.75%) |  |  |  |
| ph049d7 | 7790 (94.09%) | 489 (5.91%) |  |  |  |
| ph049d8 | 7962 (96.17%) | 317 (3.83%) |  |  |  |
| ph049d9 | 7778 (93.95%) | 501 (6.05%) |  |  |  |
| ph049d10 | 8128 (98.18%) | 151 (1.82%) |  |  |  |
| ph049d11 | 8090 (97.72%) | 189 (2.28%) |  |  |  |
| ph049d12 | 7403 (89.42%) | 876 (10.58%) |  |  |  |
| ph049d13 | 8015 (96.81%) | 264 (3.19%) |  |  |  |
|  | 1=Yes, n (%) | 5=No, n (%) |  |  |  |
| mh002 | 4770 (57.62%) | 3509 (42.38%) |  |  |  |
| mh007 | 5398 (65.2%) | 2881 (34.8%) |  |  |  |
| mh010 | 6284 (75.9%) | 1995 (24.1%) |  |  |  |
| mh013 | 5727 (69.18%) | 2552 (30.82%) |  |  |  |
| mh014 | 7412 (89.53%) | 867 (10.47%) |  |  |  |
| mh015 | 7377 (89.1%) | 902 (10.9%) |  |  |  |


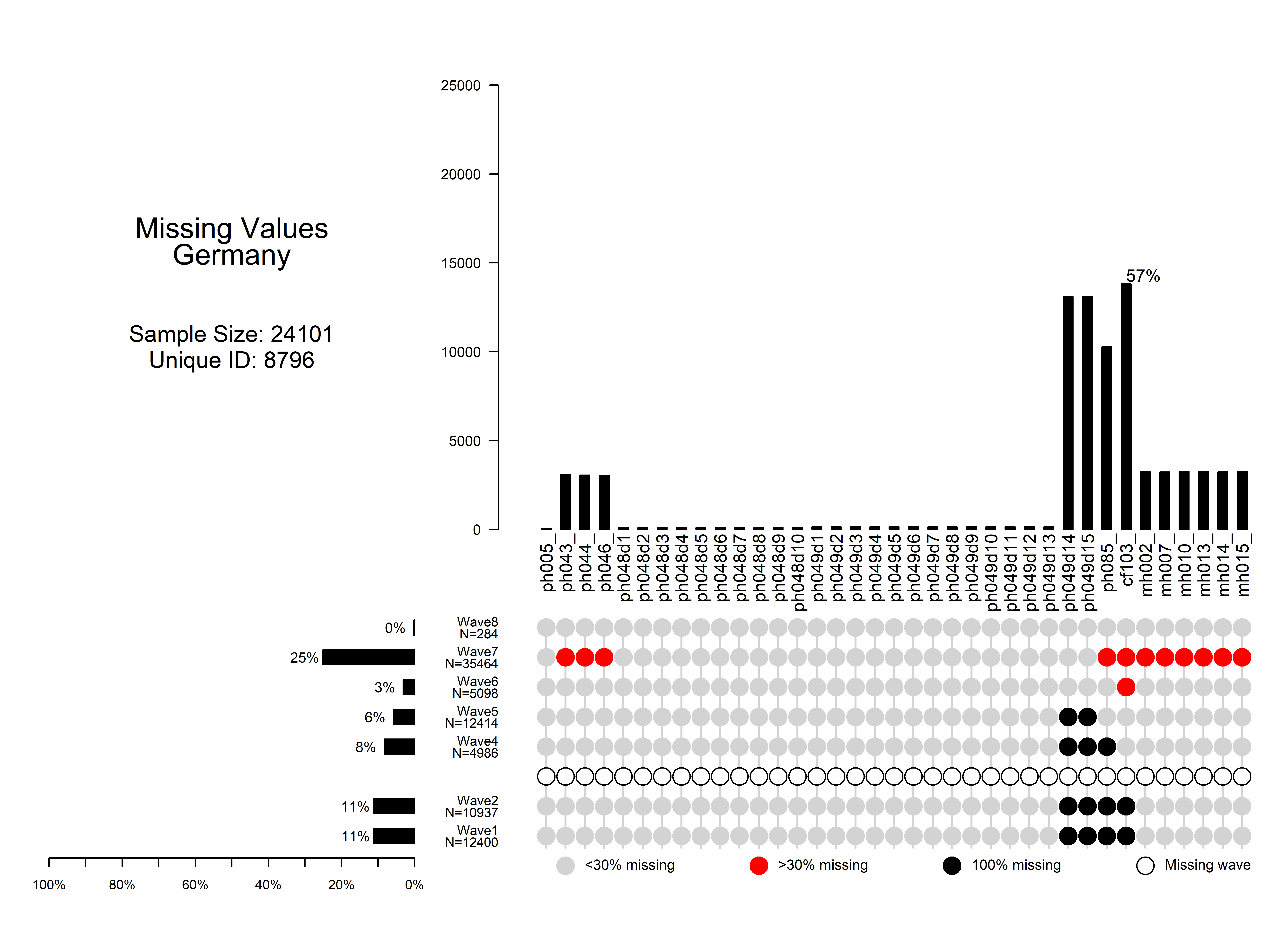


**Fig 4. Overview of missing observations in SHARE Germany.**

**Table 6. Response Frequencies for SHARE Italy after imputation (N = 8063).**

| **Item** | **Response options** |  |  |  |  |
| --- | --- | --- | --- | --- | --- |
|  | 1=Severely limited,  n (%) | 2=Limited, but not severely, n (%) | 3=Not limited, n (%) |  |  |
| ph005 | 4783 (59.32%) | 2104 (26.09%) | 1176 (14.59%) |  |  |
|  | 1=Excellent, n (%) | 2=Very good, n (%) | 3=Good, n (%) | 4=Fair, n (%) | 5=Poor, n (%) |
| ph043 | 710 (8.81%) | 1325 (16.43%) | 4230 (52.46%) | 1384 (17.16%) | 414 (5.13%) |
| ph044 | 503 (6.24%) | 1136 (14.09%) | 4097 (50.81%) | 1682 (20.86%) | 645 (8%) |
| ph046 | 933 (11.57%) | 2044 (25.35%) | 3497 (43.37%) | 1250 (15.5%) | 339 (4.2%) |
|  | Not selected, n (%) | 1=Selected, n (%) |  |  |  |
| ph048d1 | 6988 (86.67%) | 1075 (13.33%) |  |  |  |
| ph048d2 | 7209 (89.41%) | 854 (10.59%) |  |  |  |
| ph048d3 | 6635 (82.29%) | 1428 (17.71%) |  |  |  |
| ph048d4 | 5492 (68.11%) | 2571 (31.89%) |  |  |  |
| ph048d5 | 6607 (81.94%) | 1456 (18.06%) |  |  |  |
| ph048d6 | 5609 (69.56%) | 2454 (30.44%) |  |  |  |
| ph048d7 | 7171 (88.94%) | 892 (11.06%) |  |  |  |
| ph048d8 | 6901 (85.59%) | 1162 (14.41%) |  |  |  |
| ph048d9 | 6068 (75.26%) | 1995 (24.74%) |  |  |  |
| ph048d10 | 7703 (95.54%) | 360 (4.46%) |  |  |  |
| ph049d1 | 7391 (91.67%) | 672 (8.33%) |  |  |  |
| ph049d2 | 7760 (96.24%) | 303 (3.76%) |  |  |  |
| ph049d3 | 7418 (92%) | 645 (8%) |  |  |  |
| ph049d4 | 7822 (97.01%) | 241 (2.99%) |  |  |  |
| ph049d5 | 7681 (95.26%) | 382 (4.74%) |  |  |  |
| ph049d6 | 7755 (96.18%) | 308 (3.82%) |  |  |  |
| ph049d7 | 7254 (89.97%) | 809 (10.03%) |  |  |  |
| ph049d8 | 7656 (94.95%) | 407 (5.05%) |  |  |  |
| ph049d9 | 7404 (91.83%) | 659 (8.17%) |  |  |  |
| ph049d10 | 7786 (96.56%) | 277 (3.44%) |  |  |  |
| ph049d11 | 7752 (96.14%) | 311 (3.86%) |  |  |  |
| ph049d12 | 7237 (89.76%) | 826 (10.24%) |  |  |  |
| ph049d13 | 7627 (94.59%) | 436 (5.41%) |  |  |  |
|  | 1=Yes, n (%) | 5=No, n (%) |  |  |  |
| mh002 | 5083 (63.04%) | 2980 (36.96%) |  |  |  |
| mh007 | 5739 (71.18%) | 2324 (28.82%) |  |  |  |
| mh010 | 5050 (62.63%) | 3013 (37.37%) |  |  |  |
| mh013 | 5206 (64.57%) | 2857 (35.43%) |  |  |  |
| mh014 | 6575 (81.55%) | 1488 (18.45%) |  |  |  |
| mh015 | 6318 (78.36%) | 1745 (21.64%) |  |  |  |


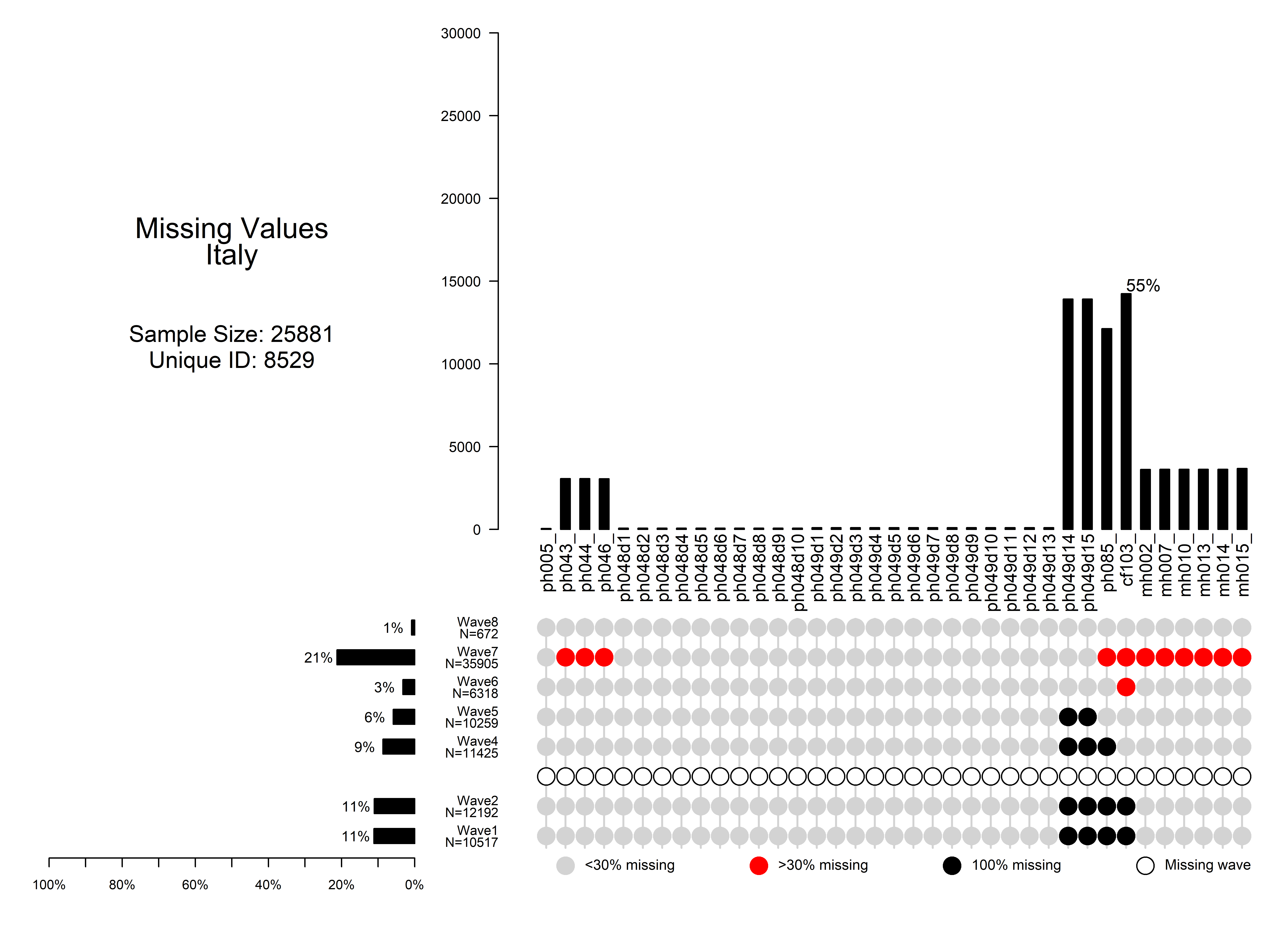


**Fig 5. Overview of missing observations in SHARE Italy.**
