# Supplementary figures and images for: Development of a functioning metric for the ageing population using data from the survey of health, ageing and retirement in Europe (SHARE)
Source: PLoS One. 2025 Apr 24;20(4):e0320068. doi: 10.1371/journal.pone.0320068 (PMC12021154; doi:10.1371/journal.pone.0320068)

# S1 Fig. Local Item Dependencies at Start.


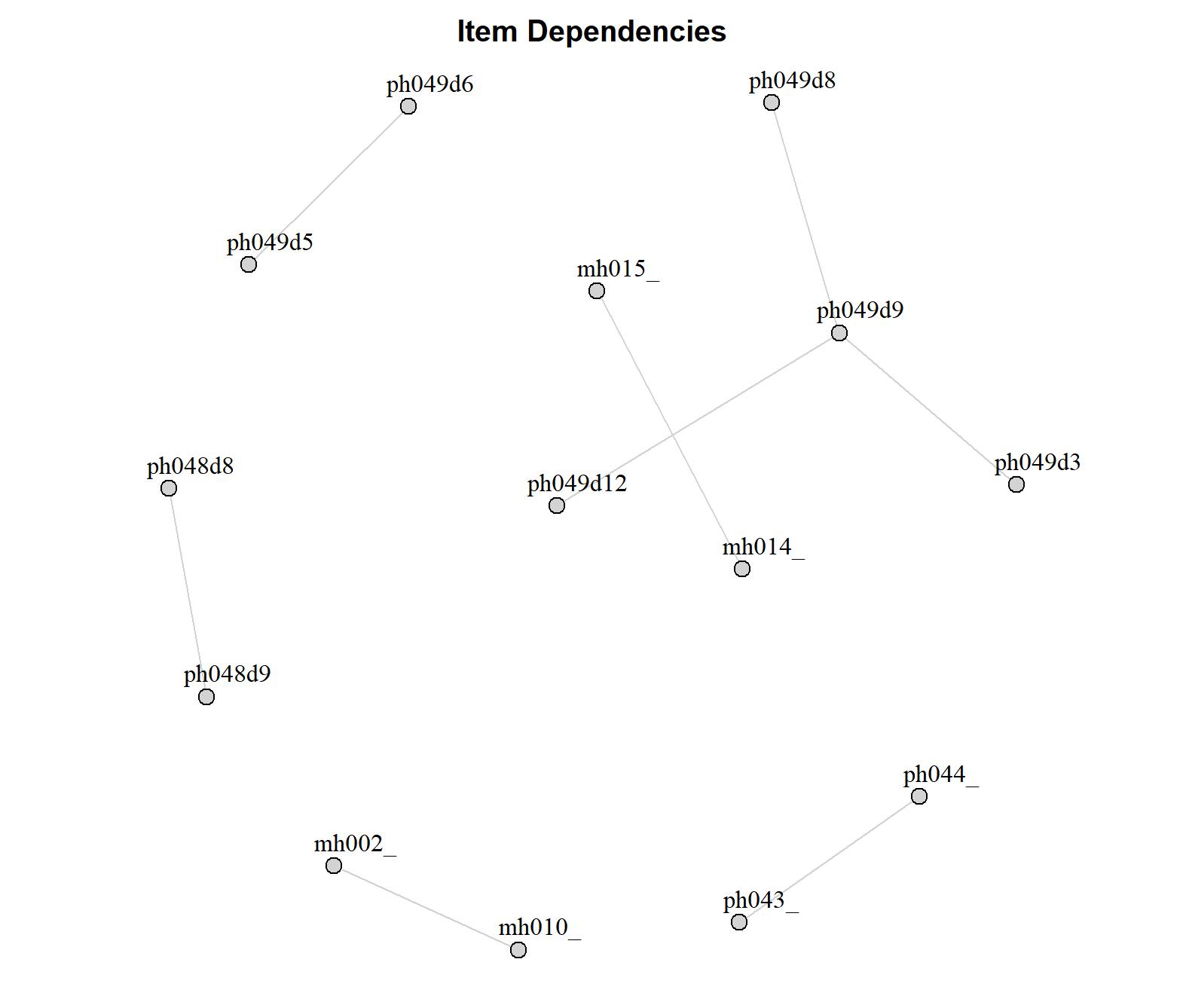

Supplement: S1 Fig — (DOCX) [file pone.0320068.s003.docx]
